# Emergence of Antifungal Resistant Subclades in the Global Predominant Phylogenetic Population of Candida albicans

**DOI:** 10.1128/spectrum.03807-22

**Published:** 2023-01-26

**Authors:** Jie Gong, Xin-Fei Chen, Xin Fan, Juan Xu, Han Zhang, Ruo-Yu Li, Sharon C-A Chen, Fanrong Kong, Shu Zhang, Zi-Yong Sun, Mei Kang, Kang Liao, Da-Wen Guo, Zhe Wan, Zhi-Dong Hu, Yun-Zhuo Chu, Hong-Mei Zhao, Gui-Ling Zou, Chong Shen, Yuan-Yuan Geng, Wei-Wei Wu, He Wang, Fei Zhao, Xin Lu, Li-Hua He, Gui-Ming Liu, Ying-Chun Xu, Jian-Zhong Zhang, Meng Xiao

**Affiliations:** a State Key Laboratory of Infectious Disease Prevention and Control, Collaborative Innovation Center for Diagnosis and Treatment of Infectious Diseases, National Institute for Communicable Disease Control and Prevention, Chinese Center for Disease Control and Prevention, Beijing, China; b Department of Laboratory Medicine, Sate Key Laboratory of Complex Severe and Rare Diseases, Peking Union Medical College Hospital, Chinese Academy of Medical Sciences, Beijing, China; c Beijing Key Laboratory for Mechanisms Research and Precision Diagnosis of Invasive Fungal Diseases, Beijing, China; d Department of Infectious Diseases and Clinical Microbiology, Beijing Institute of Respiratory Medicine and Beijing Chao-Yang Hospital, Capital Medical University, Beijing, China; e Department of Dermatology, Beijing University First Hospital, Beijing, China; f Beijing Key Laboratory of Molecular Diagnosis on Dermatoses, Beijing, China; g Research Center for Medical Mycology, Beijing University, Beijing, China; h Centre for Infectious Diseases and Microbiology Laboratory Services, Institute of Clinical Pathology and Medical Research, New South Wales Health Pathology, Westmead Hospital, University of Sydney, Sydney, New South Wales, Australia; i Department of Clinical Laboratory, Tongji Hospital, Tongji Medical College, Huazhong University of Science and Technology, Wuhan, Hubei, China; j Department of Laboratory Medicine, West China Hospital, Sichuan University, Chengdu, Sichuan, China; k Department of Clinical Laboratory, First Affiliated Hospital of Sun Yat-Sen University, Guangzhou, China; l Department of Clinical Laboratory, The First Affiliated Hospital of Harbin Medical University, Harbin, Heilongjiang, China; m Department of Clinical Laboratory, Tianjin Medical University General Hospital, Tianjin, China; n Department of Clinical Laboratory, The First Hospital of China Medical University, Shenyang, Liaoning, China; o Department of Clinical Laboratory, The People's Hospital of Liaoning Province, Shenyang, Liaoning, China; p Department of Clinical Laboratory, The Fourth Affiliated Hospital of Harbin Medical University, Harbin, Heilongjiang, China; q Center for Statistical Science, and Department of Industrial Engineering, Tsinghua University, Beijing, China; r Department of Dermatology, the Fifth People's Hospital of Hainan Province, Haikou, Hainan, China; s Dynamiker Sub-center of Beijing Key Laboratory for Mechanisms Research and Precision Diagnosis of Invasive Fungal Disease, Tianjin, China; t Beijing Agro-Biotechnology Research Center, Beijing Academy of Agriculture and Forestry Sciences, Beijing, China; University of Michigan

**Keywords:** *Candida albicans*, phylogenetic population, azole-resistance, Erg11p, Tac1p, azole-nonsusceptibility

## Abstract

Candida albicans remains the most common species causing invasive candidiasis. In this study, we present the population structure of 551 global C. albicans strains. Of these, the antifungal susceptibilities of 370 strains were tested. Specifically, 66.6% of the azole-nonsusceptible (NS)/non-wild-type (NWT) strains that were tested belonged to Clade 1. A phylogenetic analysis, a principal components analysis, the population structure, and a loss of heterozygosity events revealed two nested subclades in Clade 1, namely, Clade 1-R and Clade 1-R-α, that exhibited higher azole-NS/NWT rates (75.0% and 100%, respectively). In contrast, 6.4% (21/326) of the non-Clade 1-R isolates were NS/NWT to at least 1 of 4 azoles. Notably, all of the Clade 1-R-α isolates were pan-azole-NS/NWT that carried unique A114S and Y257H double substitutions in Erg11p and had the overexpression of ABC-type efflux pumps introduced by the substitution A736V in transcript factor Tac1p. It is worth noting that the Clade 1-R and Clade 1-R-α isolates were from different cities that are distributed over a large geographic span. Our study demonstrated the presence of specific phylogenetic subclades that are associated with antifungal resistance among C. albicans Clade 1, which calls for public attention on the monitoring of the future spread of these clones.

**IMPORTANCE** Invasive candidiasis is the most common human fungal disease among hospitalized patients, and Candida albicans is the predominant pathogen. Considering the large number of infected cases and the limited alternative therapies, the azole-resistance of C. albicans brings a huge clinical threat. Here, our study suggested that antifungal resistance in C. albicans could also be associated with phylogenetic lineages. Specifically, it was revealed that more than half of the azole-resistant C. albicans strains belonged to the same clade. Furthermore, two nested subclades of the clade exhibited extremely high azole-resistance. It is worth noting that the isolates of two subclades were from different cities that are distributed over a large geographic span in China. This indicates that the azole-resistant C. albicans subclades may develop into serious public health concerns.

## INTRODUCTION

Candida albicans is a common commensal organism of the human skin and gut. Worldwide, it may be the most common pathogenic cause of invasive fungal diseases (IFDs), which are often severe and life-threatening (1, 2). The treatment options for IFDs are quite limited. Only four classes of antifungal agents, namely, azoles, echinocandins, polyenes, and nucleoside analogues, are available for IFD therapy at the present time, and the loss of efficacy for any antifungal agent due to the emergence of resistance raises substantial clinical concerns (3, 4). Although echinocandin therapy is becoming more important for invasive candidiasis, azole antifungals remain one of the most commonly used treatment options (3, 5). A systematic investigation report pointed out that azoles accounted for 71.5% of the antifungal drugs that were used for fungal bloodstream infections in China (6).

Azole-resistance in *Candida* species is often related to the presence of mutations in the *ERG11* gene, which encodes lanosterol 14-α-demethylase (Erg11p), which is the main target of azoles (7, 8). Studies have revealed that certain mutations in *ERG11* that result in amino acid substitutions compromise the drug-binding abilities of Erg11p and consequently reduce drug efficacy (9–13). Azole-resistance in *Candida* species may also be caused by the overexpression of *ERG11* or of genes encoding drug efflux pumps (8). Moreover, drug resistance in C. albicans can also be related to a loss-of-heterozygosity (LOH) and aneuploidy events (4, 7).

Previous genomics-based studies using traditional multilocus sequence typing (MLST) and whole-genome sequencing (WGS) have identified more than 18 genetic populations within C. albicans (14–17). Of note, the cluster identification results of MLST and WGS have been similar (16). Here, we carried out a population-based genetic study of 551 C. albicans strains, including 181 previously published global C. albicans genomes (16) and 370 isolates that cause causing invasive fungal infections that were collected from 33 cities in China using WGS. Our analysis revealed that most (33/54, 61.1%) of the azole-nonsusceptible (NS)/non-wild-type (NWT) C. albicans isolates were assigned to phylogenetic Clade 1-R within the previously recognized Clade 1, which is the predominant phylogenetic population that is widely found around the world. Furthermore, the subclade Clade 1-R-α, in which 100% of isolates were NS/NWT to all azole antifungals, was identified.

## RESULTS

### Clade 1 predominated worldwide, with other clones being more geographically diverged.

A total of 741,793 single nucleotide polymorphisms (SNPs) were identified from the 551 genomes for further analysis. For the 370 clinical strains that were collected in this study, a total of 634 Gb of high-quality genomic sequence was generated, with a mean sequencing depth of 142× per isolate (range 66 to 399) (Fig. S2). The mean sequencing depth of the previously published 181 global genomes was 108× (ranging from 6× to 250×) (Fig. S2) (16).

**FIG 1:**
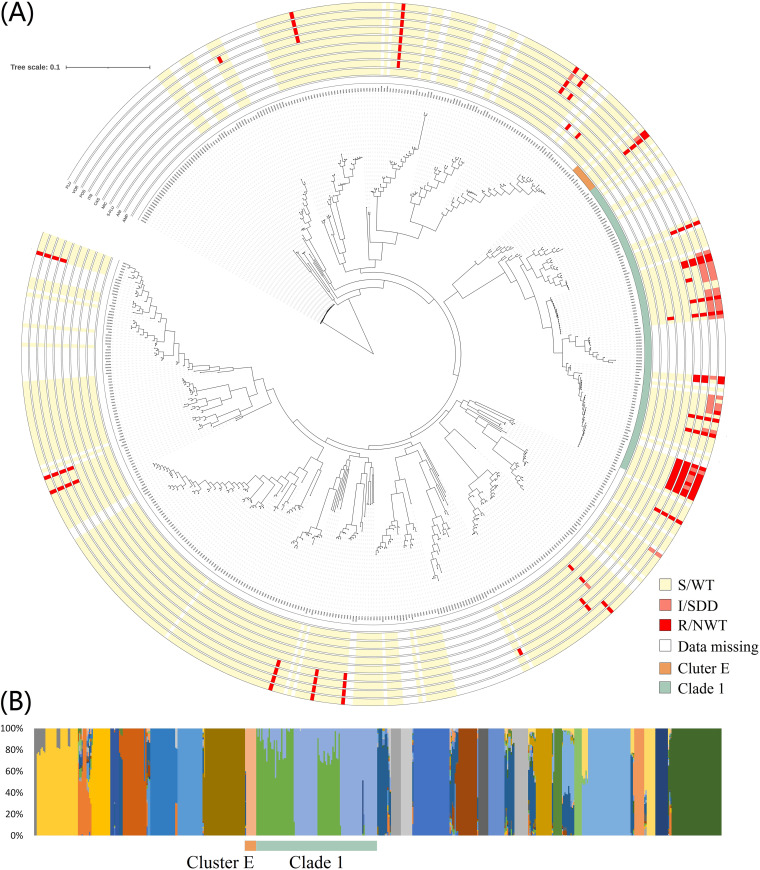
Phylogenetic relationships and population structures of C. albicans. The figure contains information on 370 C. albicans clinical isolates that were collected in this study as well as on 181 global C. albicans genomes. (A) A maximum likelihood phylogenetic tree was generated, based on the whole-genome SNPs, and the midpoint rooting method was used to root the tree. Clade 1 and Cluster E (the closest clade to Clade 1) isolates are highlighted with different colors. The susceptibility to antifungals was shown in outer circles, from outside to inside: fluconazole, voriconazole, itraconazole, posaconazole, caspofungin, micafungin, 5-flucytosine, anidulafungin and amphotericin B. (B) Population structure of C. albicans. The number of populations (K = 28) is shown. Clade 1 and the adjacent Cluster E are highlighted.

To elucidate the phylogenetic characteristics of the C. albicans isolates, we carried out a maximum likelihood (ML) analysis, A principal components analysis (PCA), an ADMIXTURE analysis, and the results are shown in Fig. 1 and in Fig. S5. The lowest cross-validation error was found at K = 28 (Fig. S8A). The Clade 1 to 18 and Cluster A to E nomenclatures were used, as per previous MLST and WGS studies (14–16).

It was revealed that more than half (*n* = 375, 68.0%) of the 551 strains could be classified into 14 previously known clades (16), mainly Clade 1 (*n* = 97, 17.6%), Clade 4 (*n* = 43, 7.8%) and Clade 18 (*n* = 30, 8.1%) (Tables S1 and S2). Of note, Clade 1 was the largest clonal population, both among the 181 previously reported global isolates (*n* = 40, 22.1%) (16) and among the 370 clinical isolates that were collected in this study (*n* = 57, 15.4%). However, none of the Chinese isolates belonged to Clade 13 or to Clade 2, which were the second and third largest clonal populations identified in the 181 global strains (accounting for 19.3% [35/181] and 14.9% [27/181] of the collection, respectively) (16), indicating the diversity of the phylogenetic population across geographic regions. In addition, 3.6% (20/557) and 1.6% (9/557) of the strains were assigned to the previously proposed Cluster A and Cluster E, respectively (Tables S1 and S2; Fig. S3).

Moreover, several novel clonal populations were identified by our analysis, and we named them groups 1 to 21 (Tables S1 and S2). These groups consisted of 19.2% (106/551) of the isolates collected, with group 4 being the largest (3.4%, 19/551). The newly designated groups also included four global strains that had not been assigned to previously known clades or clusters by Ropars et al. (Tables S1 and S2) (16). Finally, there were 30 isolates that had not been assigned to any of the above-mentioned clades, clusters, or groups (Tables S1 and S2).

### LOH events were frequent and revealed specific phylogenetic subclades in Clade 1.

**FIG 2:**
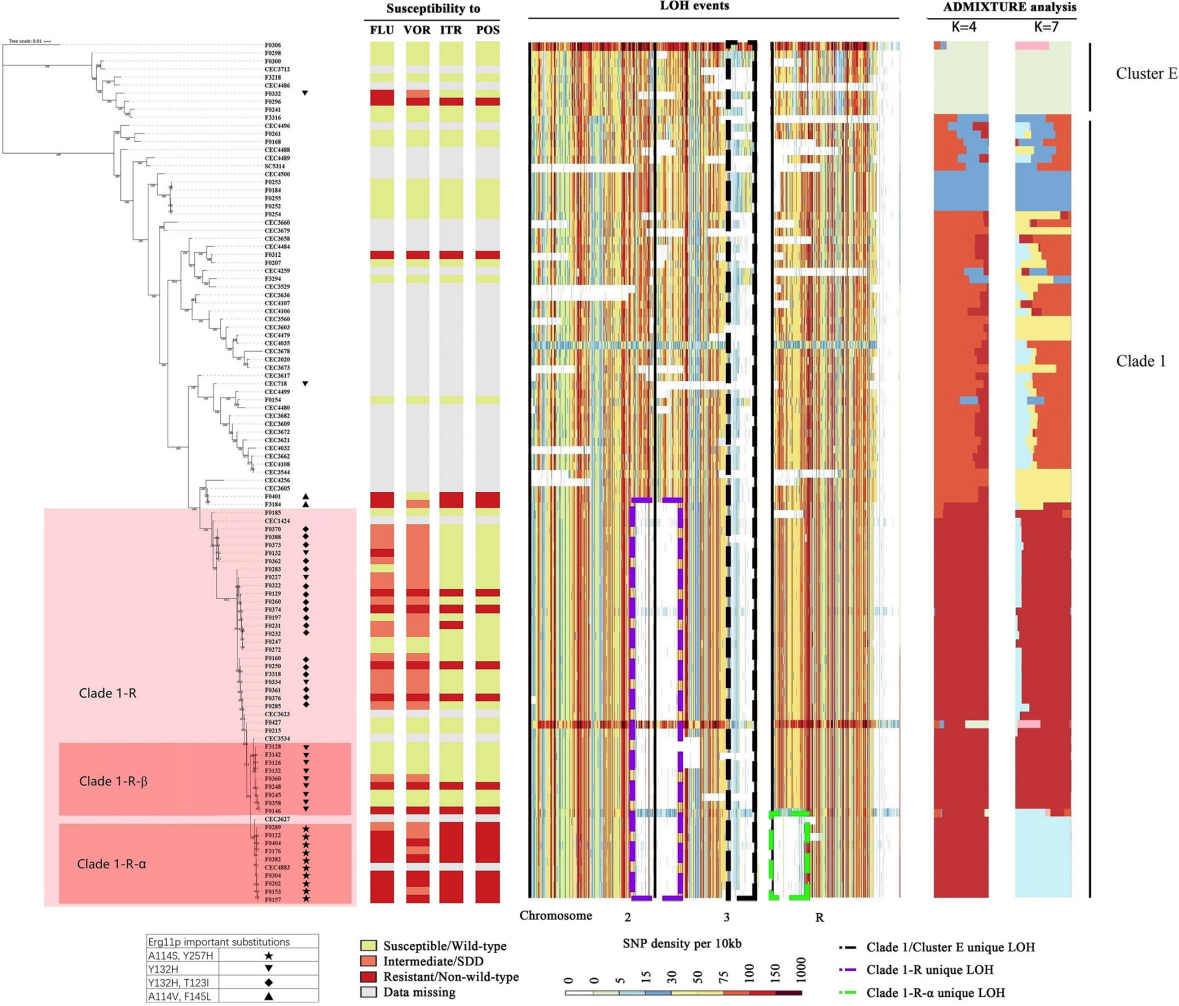
Phylogenetic relationships, azole *in vitro* susceptibilities, important Erg11p substitutions, loss-of-heterozygosity (LOH) events, and population structures of the Clade 1 and Cluster E C. albicans isolates in this study. (Left panel) A maximum likelihood phylogenetic tree was generated, based on the whole-genome SNPs and using strain F0306 as an outgroup (assigned to group 6, which is adjacent to Clade 1 and Cluster E). Clade 1-R, Clade 1-R-α, and Clade 1-R-β are highlighted in a pink background color. (Middle panel) In the LOH analysis, the density of heterozygous SNPs was calculated using C. albicans SC5314 as a reference and is shown in 10 kb windows. Each row represents a corresponding strain in the maximum likelihood tree. The black box indicates the LOH event identified in Clade 1-R, as in a previous study by Ropars et al. (16), whereas the purple and green boxes indicate unique LOH events that are newly identified in Clade 1-R and Clade 1-R-α, respectively. (Right panel) ADMIXTURE population structure analysis (K = 4 and K = 7). Information regarding susceptibility to azoles and important Erg11p substitutions is also shown.

It has been established that phenotypic diversity in diploid species, such as C. albicans, can arise rapidly through LOH. Numerous LOH events were detected in our study. These included two previously reported, major, ancient LOH events that were identified within Clade 1 and Clade E (indicated by a black dotted box in Fig. 2) as well as Clade 4 (indicated by a black dotted box in Fig. S4), respectively (16). Strain-specific LOH events were also frequent (Fig. 2; Fig. S4, horizontal light-colored stripes).

Of note, three additional, recent LOH events were found in the Clade 1 isolates. The first two occurred in 77.2% (44/51) of the Clade 1 isolates in the present study and were identified in five global isolate genomes (indicated by a purple dotted box in Fig. 2) that were found on the terminal end of the C. albicans chromosomes 2 and 3. We named this subclade Clade 1-R. The third LOH event occurred on the terminal end of chromosome R and was found in a further subset of isolates in Clade 1-R (indicated by a green dotted box in Fig. 2). This LOH event was observed in 9 isolates (17.6% in Clade 1). In addition, one isolate from the global collection that was also originally isolated in China was assigned to this subclade (16, 18). This unique subclade of Clade 1-R was named Clade 1-R-α.

### Phylogenetic footprints further support the presence of Clade 1-R and Clade 1-R-α.

The ML analysis and ADMIXTURE were carried out within Clade 1 and Cluster E (a close cluster of Clade 1) to further observe the genetic differentiation of the subclades. The ML tree of the Clade 1 and Cluster E isolates revealed that all Clade 1-R and Clade 1-R-α isolates were clustered together (Fig. 2). Using ADMIXTURE, when K = 2, a division was found between Clade 1 and Cluster E. The lowest cross-validation errors were found at K = 4 and K = 7 (Fig. S8B). When K ≥ 3, differentiation of Clade 1-R from Clade 1 was observed (Fig. 2; Fig. S11). When K ≥ 7, differentiation of Clade 1-R-α from Clade 1-R was observed (Fig. 2; Fig. S11). The PCA results also indicated the presence of subclade 1-R and Clade 1-R-α (Fig. S5). Moreover, all of the Clade 1-R-α isolates inherited unique key *ERG11* and *TAC1* mutations as well as the corresponding resistance phenotypes (see detailed results in the following sections), which supports the nomenclature of this independent subclade.

The average numbers of SNPs in all pairwise comparisons of strains within the whole collection (*n* = 551), the Clade 1 isolates (*n* = 97), the Clade 1-R isolates (*n* = 49) and the Clade 1-R-α isolates (*n* = 10) were 1018, 75, 58, and 41 nucleotides, respectively. The values of nucleotide diversity (π), were, in ascending order, Clade 1-R-α (0.002783), Clade 1-R (0.002877), Clade 1 (0.003081), and all 551 isolates (0.006425) (detailed in Table S2). The maximum and minimum numbers of SNPs separating any two isolates from within clades/subclades were 1515 and 22, respectively (detailed in Table S2). Fig. S10 shows a heat map of the pairwise SNP distances, based on the absolute distance of the SNPs of Clade 1 and the adjacent Cluster E.

**FIG 3:**
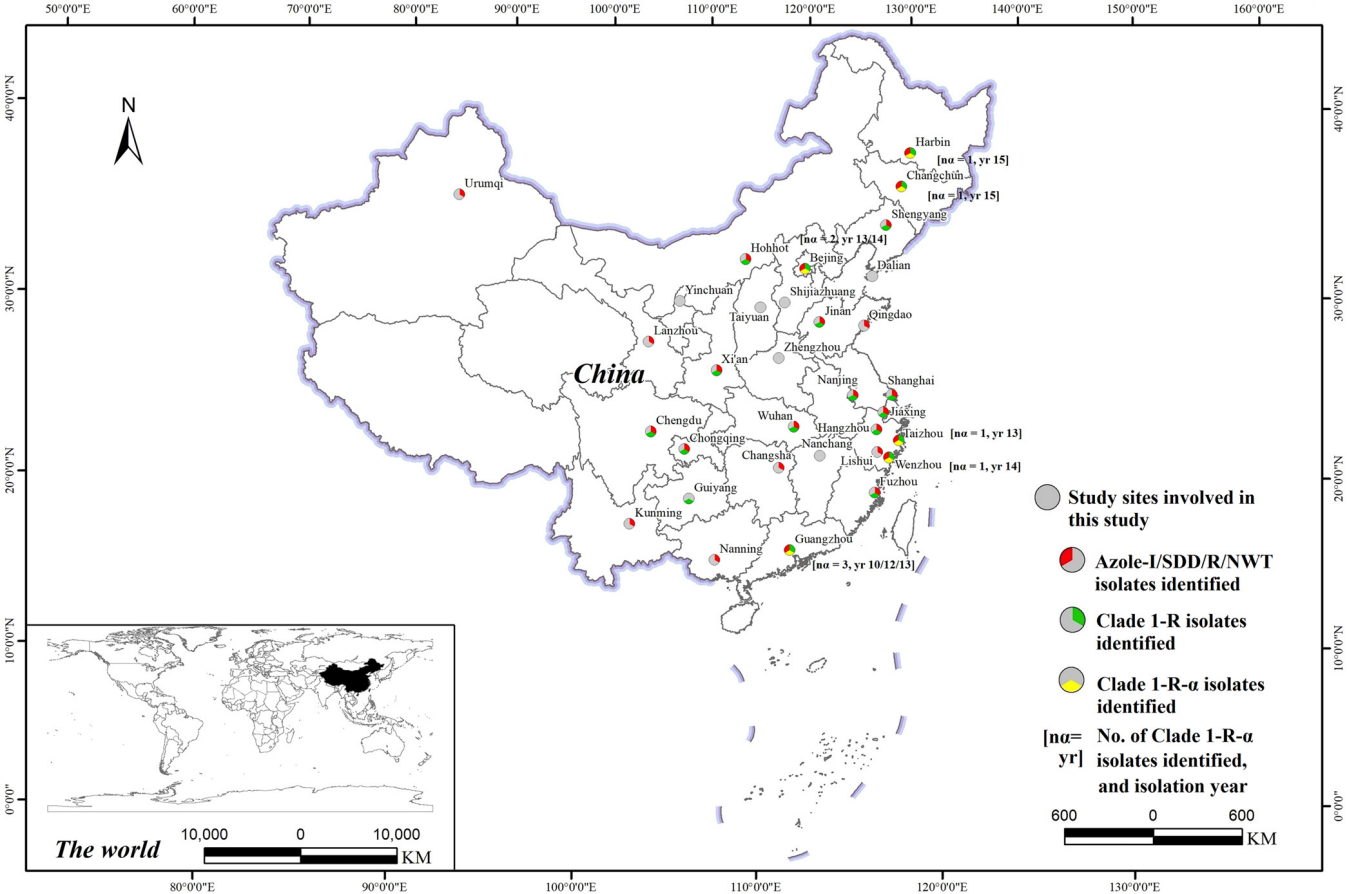
Geographic distribution of the 33 cities from which the 370 C. albicans isolates in this study were collected (gray circles). The map was generated using ArcGIS Desktop (version 10.6, ESRI, Redlands, USA). Information on whether the azole-NS/NWT isolates and isolates belonging to Clade 1-R and Clade 1-R-α were identified in each city is also illustrated.

Among the isolates collected in this study, the 44 isolates assigned to Clade 1-R were from 19 different cities, and the 9 isolates assigned to Clade 1-R-α were from 6 different cities (Fig. 3; Table S1). The Clade 1-R-α strains were isolated from blood (5/9, 55.6%), ascitic fluid (1/9, 11.1%), venous catheters (1/9, 11.1%), tissue (1/9, 11.1%), and pus (1/9, 11.1%). There were three isolates detected in a single hospital (F0122, F0153, and F0157), and these displayed high genome similarity (between 38 and 42 pairwise different SNPs). However, these strains were isolated in three different years (2010, 2012, and 2013) and from different departments (nephrology department, department of respiratory medicine, and intensive care unit). Therefore, no obvious clonal spread of Clade 1-R-α was identified in hospital settings in the current study, which excluded the possibility of nosocomial outbreaks.

### Clade 1-R and Clade 1-R-α were associated with azole-resistance.

Overall, 54 (14.6%) isolates were classified as I/SDD/R/NWT to at least one azole. Additionally, 13.2% (*n* = 49), 13.2% (*n* = 49), 8.6% (*n* = 32), and 8.6% (*n* = 32) of the isolates were I/SDD/R/NWT to fluconazole, voriconazole, itraconazole, and posaconazole, respectively. In addition, 92.6% (50/54) of the isolates were I/SDD/R/NWT to more than two azoles. Five isolates were NWT to 5-flucytosine, and two isolates were I/R to the echinocandins, but none were resistant to amphotericin B.

All of the Clade 1 isolates in this study were classified as S/WT to echinocandins and amphotericin B (Tables S1 and S2). However, among the Clade 1 isolates, especially those belonging to Clade 1-R and Clade 1-R-α, there were high azole-NS/NWT rates (Table S2). Generally, Clade 1 contained 66.6% (36/54) of the azole-NS/NWT strains, which in turn accounted for 63.2% (36/57) of the isolates belonging to this clade. Notably, 75.0% (33/44) of the Clade 1-R isolates were NS/NWT to at least one of the four azoles, including 50.0% (22/44) that were NS/NWT to all four azoles (Fig. 2; Table S2). Moreover, all nine of the isolates assigned to Clade 1-R-α were NS/NWT to all of the azole drugs. In contrast, 6.4% (21/326) of the non-Clade 1-R isolates were NS/NWT to at least one of the four azoles. There was one 5-flucytosine-NWT strain assigned to Clade 1-R (Tables S1 and S2).

Genes on the unique LOH event regions of Clade 1-R and Clade 1-R-α were identified (detailed in Table S4). Although the genes *ERG4*, *ERG25*, and *ERG27*, which are involved in ergosterol biosynthesis, are located in this region, we did not find strong evidence for a direct association between LOH events and azole-resistance. A read depth analysis did not identify any significant aneuploidy phenomena as contributing to azole-resistance.

### Important *ERG11* gene mutations led to azole-resistance.

The mutations in 12 selected genes that were previously reported to be associated with antifungal resistance are detailed in Table S1.

There were 29 nonsynonymous mutations found in the *ERG11* gene among the 370 strains (Table S1). Previous publications related to the functional verification of *ERG11* gene mutations were reviewed, and we found that 9 of the 29 mutations identified in this study (resulting in amino acid substitutions A114S, T123I, Y132H, K143R, F145L, Y257H, L301S, S405F, and G448E) were previously verified to cause an increase in the azole MICs (Table S5) (9–12). Among the 54 azole-NS/NWT isolates, Y132H was the most common substitution found (occurring in 48.1% of the azole-NS/NWT isolates, 26/54), and this was followed by T123I (33.3%, 18/54), A114S (16.7%, 9/54), and Y257H (16.7%, 9/54), whereas the prevalence of F145L (3.7%, 2/54), K143R (1.9%, 1/54), L301S (1.9%, 1/54), S405F (1.9%, 1/54), or G448E (1.9%, 1/54) was rare (Table 1; Table S1). In addition, there was a novel substitution, L301S, that was observed in only one azole-NS/NWT isolate (Table 1; Table S1). Using a heterologous expression model of S. cerevisiae (19), we also verified that all nine of the important substitutions mentioned above could increase the fluconazole MICs, including the novel L301S (Table S5; Fig. S9). Overall, these nine important nucleotide mutations in the *ERG11* gene were responsible for 70.3% (*n* = 38) of the 54 azole-NS/NWT cases.

**TABLE 1:** Phylogenetic characteristics of 54 azole-NS/NWT isolates and associations with Erg11p substitutions

Phylogenetic clades	Important Erg11p substitutions	Corresponding *ERG11* gene mutations	Other Erg11p substitutions	No. of isolates that were azole-nonsusceptible (%)
Clade 1/Cluster E azole nonsusceptible isolates (*n* = 38)				
Clade 1-R-α	A114S, Y257H	G340T, T769C	None	9 (16.7)
Clade 1-R-β	Y132H	T394C	G465S	3 (5.6)
Clade 1-R (non-α/β)	Y132H, T123I	T394C, C368T	None	17 (31.5)
	Y132H, T123I, K143R	T394C, C368T	None	1 (1.9)
	Y132H, G448E	T394C, G1343A	None	3 (5.6)
Clade 1 (non-1-R)	F145L	T433C	A114V	2 (3.7)
	None	None	None	1 (1.9)
Cluster E	Y132H, S405F	T394C, C1214T	None	1 (1.9)
	None	None	None	1 (1.9)
Non-Clade 1/Cluster E azole nonsusceptible isolates (*n* = 16)				
Clade 4	L301S	T902C	V488I, E266D	1 (1.9)
Group 4	Y132H	T394C	D153E	1 (1.9)
Clade 9	None	None	None	1 (1.9)
Clade 11	None	None	None	1 (1.9)
Clade 15	None	None	None	2 (3.7)
Clade 18	None	None	None	2 (3.7)
Cluster A	None	None	None	1 (1.9)
Group 6	None	None	None	1 (1.9)
Group 10	None	None	None	1 (1.9)
Group 11	None	None	None	2 (3.7)
Group 19	None	None	None	1 (1.9)
Group 20	None	None	None	1 (1.9)
Singleton	None	None	None	1 (1.9)

### *ERG11* genotypes were associated with C. albicans phylogenetic clades and differential azole susceptibility.

Notably, all 33 azole-NS/NWT isolates within Clade 1-R carried at least one important substitution in Erg11p (Table 1). It was also noteworthy that all nine of the strains that were assigned to Clade 1-R-α in this study, along with the only Clade 1-R-α isolate found in the global collection, carried cross-linked A114S and Y257H substitutions in Erg11p (Table 1; Fig. 2; Table S1), whereas these two substitutions were not found in any other phylogenetic population, further supporting the phylogenetic relatedness of the Clade 1-R-α isolates.

In the ML tree, a set of Clade-1-R isolate branches occurred immediately adjacent to Clade 1-R-α (*n* = 9), and these were collectively labeled Clade 1-R-β (Fig. 2). All of the Clade 1-R-β isolates carried one important Erg11p substitution, namely, Y132H (as well as three other substitutions that do not affect azole susceptibility, namely, D116E, K128T, and G465S), which was unique among all of the C. albicans isolates collected (Table S1) and was distinct from the Erg11p mutations in Clade 1-R-α (Fig. 2). Although 66.7% (6/9) of the Clade 1-R-β isolates were azole susceptible or of the WT phenotype (Fig. 2), these isolates had obviously higher fluconazole and voriconazole MIC distributions than did many of the wild-type isolates (Fig. S6).

There were 22 isolates carrying Y132H coupled with one or more other important substitutions, including 17 with T123I, 3 with G448E, 1 with S405F, and 1 with T123I+K143R (Table 1). All of these isolates were azole-NS/NWT (Fig. 2; Table S1). In addition, except for the Y132H+S405F isolate, which belongs to Clade E, all 21 of the other isolates were found in Clade 1-R (Fig. 3).

### The overexpression of ABC-type efflux pumps further contributed to azole-resistance and was related to *TAC1* gene mutations.

**FIG 4:**
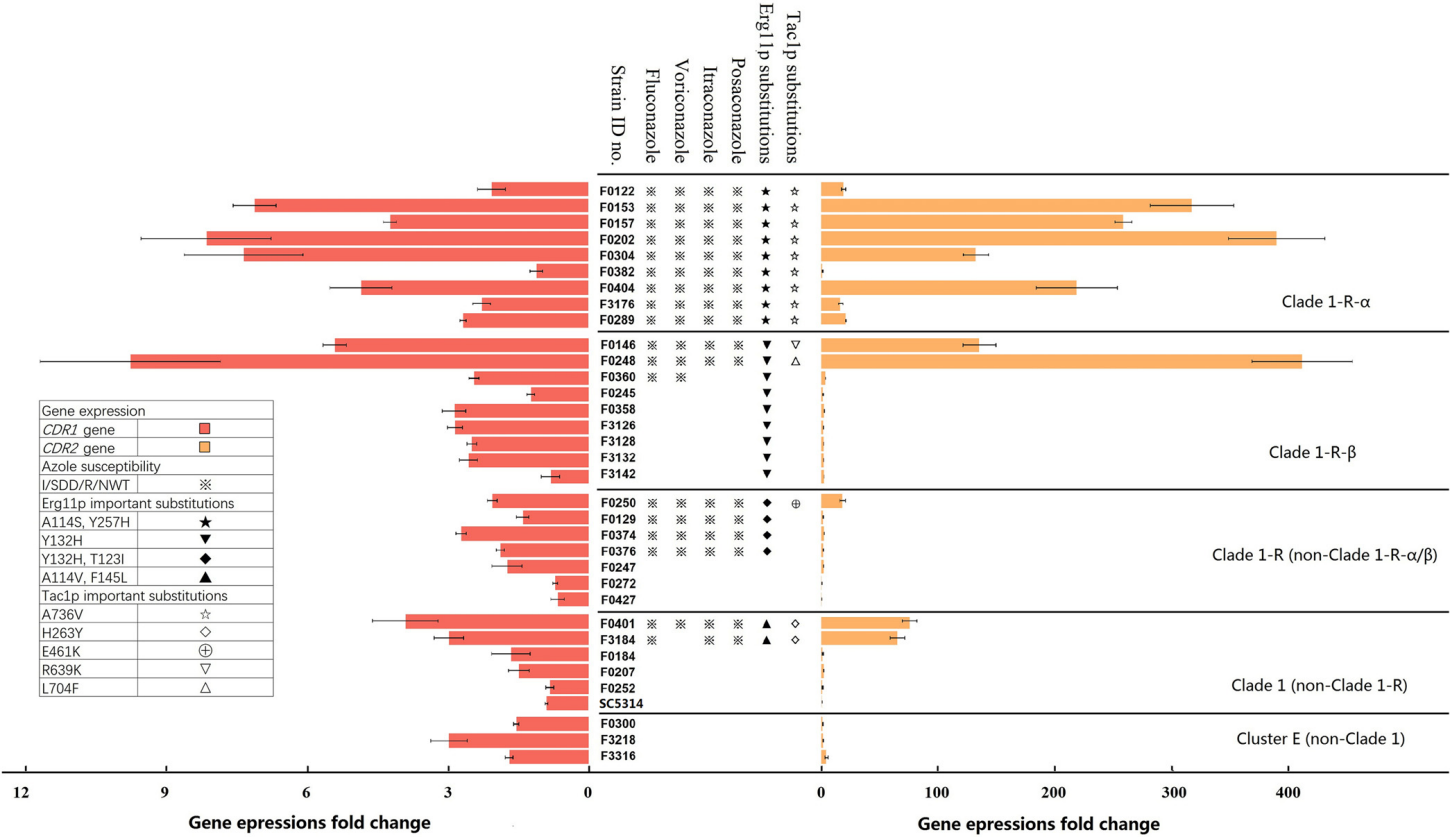
The expression fold change of the *CDR1* and *CDR2* genes in 33 selected Clade 1/Cluster E clinical isolates. The azole susceptibility phenotype and important substitutions in Erg11p and Tac1p for each strain are also shown. Symbols: ※, azole-I/SDD/R/NWT phenotypes; ★, A114S and Y257H of Erg11p; ▾, Y132H of Erg11p; ◆, Y132H and T123I of Erg11p; ▴, F145L and A114V of Erg11p; ⋆, A736V of Tac1p; ◇, H263Y of Tac1p; ⊕, E461K of Tac1p; ▽, R639K of Tac1p; and △, L704F of Tac1p.

Among the 33 Clade 1/Cluster E isolates tested, there was no significant upregulation found in the expression level of the *ERG11* gene among the azole-NS/NWT isolates. Furthermore, no difference was found in the expression level of the *CYTb* gene, which regulates respiratory status, or in the *MDR1* and *FLU1* genes, which encode two different types of drug efflux pumps with different activities between the azole-S/WT and NS/NWT isolates (Fig. 4; Table S7). However, the expression of the *CDR1* and *CDR2* genes of the Clade 1-R-α isolates was significantly higher than that of the azole-S/WT isolates, based on a *t* test (*P* < 0.005). This might suggest that Clade 1-R-α shows overexpression of ABC-type efflux pumps. Within Clade 1-R-α, the *CDR2* and *CDR1* genes were significantly upregulated in 100% (9/9; 17.2 to 390.5-fold) and 55.6% (5/9; 4.2 to 8.1-fold) of the isolates, respectively, compared with the reference strain SC5314 (Fig. 4). Moreover, all of the Clade 1-R-α isolates carried the substitution A736V in the Cdr transcription factor Tac1p. The only two Clade 1-R-β isolates (F0146 and F0248) that were NS/NWT to all four azoles also exhibited high expression levels of the *CDR1* (5.4-to 9.8-fold) and *CDR2* (136.0 to 412.2-fold) genes, which carried the Tac1p substitutions R693K and L704F, respectively, whereas no alteration was observed in the azole-S/WT Clade 1-R-β isolates. The overexpression of the *CDR2* gene was also found in a Clade 1-R azole-NS/NWT isolate (F0250, 18.8-fold) with the Tac1p substitution E461K as well as in two Clade 1 azole-NS/NWT isolates (F0401, F3184; 65.6 to 76.1-fold), both of which had the Tac1p substitution H263Y. However, no upregulation of the *CDR1* or *CDR2* gene was observed in the azole-S/WT Clade 1/Clade E isolates that were tested.

### Gene mutations related to 5-flucytosine and echinocandin susceptibility.

Eight mutations were found in the *FUR1* gene in this study, but only the R101C substitution has been proposed to affect the susceptibility to 5-flucytosine (20). One of four (25.0%) 5-flucytosine-NWT strains carried R101C, and the MICs of 50 strains carrying the R101C mutation were significantly higher than those of strains not carrying the R101C mutation (*P* < 0.001) (Fig. S7). However, we did not identify any mutations in either the *FUR1* or *FCA1* genes that could explain the increased 5-flucytosine MICs in the remaining three NWT strains. We could not find the mutation that had been reported in the *GSC1*, *GSL1*, and *GSC2* genes. No novel mutation that was related to echinocandin-I/R was found.

## DISCUSSION

Under selective pressure, lineages with specific survival advantages tend to have a greater likelihood of producing offspring and gradually increase their proportions in the population (21, 22). For clinical pathogens, survival advantages may be reflected in their pathogenicity, stress tolerance, and antimicrobial resistance.

Compared with bacteria and viruses, there have been few previous studies focused on the associations between lineages of pathogenic fungi with specific survival advantages. However, with the emergence and global spread of “superbug” fungi, such as C. auris (23, 24), researchers and clinicians would also have to consider this affair. To date, it has been revealed that the antifungal resistance of C. auris strains in different lineages were diverged (24). Similar findings have also been reported in C. albicans, the predominant human-pathogenic fungal species. Previous studies demonstrated that C. albicans Clade 1, which was the most common phylogenetic clade distributed worldwide (16, 25, 26), was associated reduced antifungal susceptibility to 5-flucytosine and terbinafine (15, 16, 25). While neither 5-flucytosine nor terbinafine was a first-line antifungal agent for the treatment of lethal invasive fungal infections. There are clear associations between both clades and specific resistance mechanisms (clades 1 and 3 with *TAC1* and *MRR1* mutations, respectively) and even between subclades and specific combinations of mutations in these genes (subclade 1c and *ERG11*-K143R and TAC1-A640V) in C. auris (27–29). However, a similar phenomenon has not yet been found in other pathogenic fungi, except in C. albicans in this study. However, more may be found in the future. In this study, we revealed for the first time that specific subclades within C. albicans Clade 1 were significantly associated with resistance to azoles, an important class of antifungal agents that is the most widely used in fungal disease treatment nowadays.

Among the C. albicans Clade 1 isolates collected in this study, the azole-NS/NWT rate was 62.1%. This was higher than that reported in a global collection (11.0%) by Odds et al. (Table S6) (25), suggesting possible geographic variation. Of note, two clonal populations within Clade 1 that were significantly associated with azole-resistance, namely, Clade 1-R and its subclade Clade 1-R-α, were found. The clustering of Clade 1-R and Clade 1-R-α was revealed via phylogeny, a PCA, the population structure, and LOH events. In addition, all Clade 1-R-α isolates exhibited an NS/NWT phenotype to the four azoles and inherited unique key substitutions in Erg11p (A114S and Y257H) and Tac1p (A736V), further supporting the designation of these isolates as a unique subclade.

It is worth noting that all 10 of the strains that were assigned to Clade 1-R-α via genomic analysis in our study (nine isolates from our collection and one from a previous global study) were isolated in China (16, 18). None of the C. albicans isolates from other geographic regions were assigned to this subclade. A review of all previous publications reporting C. albicans isolates carrying Erg11p A114S and Y257H double substitutions confirmed the above observation (Table S8). Although the genomes of these isolates were not available for phylogenetic analysis, we may propose that Clade 1-R-α originated from China. However, it is worth noting that the nine Clade 1-R-α isolates in this study were from six different cities that are distributed over a large geographic span, from Harbin in Northeast China to Guangzhou in South China, which are separated by more than 3,400 km. The wide geographic distribution of this clone indicated that it may develop into a serious public health concern.

Efforts have also been made to illustrate the mechanisms that are responsible for azole-NS/NWT among C. albicans strains in China. A genomic analysis identified eight previously reported and one new important amino acid substitutions in Erg11p, and the effects of these substitutions were also functionally verified in a S. cerevisiae heterologous expression model. Over 70% of the azole-NS/NWT isolates, including all of the azole-NS/NWT isolates in Clade 1-R and Clade 1-R-α, carried these important substitutions. In addition, the *ERG11* genotypes were associated with the C. albicans phylogenetic clades. Notably, all of the strains assigned to Clade 1-R-α carried an A114S and Y257H double substitution in Erg11p. The A114 locus in Erg11p is near the substrate channel, and the A114S substitution decreases the binding affinity between the azole molecule and the target (30). Y257 is located in the G-helix of Erg11p, and the Y257H substitution changes the amino acid residue from a neutral, uncharged tyrosine to a basic, charged histidine, which may alter channel 2 and may prevent fluconazole from accessing the active site (10). Both A114S and Y257H have been verified to increase the azole MICs (9, 10). However, isolates in Clade 1-R-β, a subclade of isolates that is phylogenetically adjacent to Clade 1-R-α, had only one key substitution (Y132H) in Erg11p and exhibited modest increases in its azole MICs (9, 22, 26). The substitution Y132H was also found in all of the other Clade 1-R azole-NS/NWT isolates that do not belong to Clade 1-R-α or Clade 1-R-β, but these isolates carried additional amino acid substitutions (T123I or G448E) in their Erg11p sequences.

Potential molecular mechanisms other than the Erg11p substitution were also investigated. At the gene expression level, no obvious changes were found in the *ERG11*, *MDR1*, *FLU1*, or *CYTb* genes. However, the significant overexpression of ABC-type efflux pumps was observed in the azole-NS/NWT isolates. Of note, all of the Clade 1-R-α isolates displayed significant overexpression of ABC-type efflux pumps and carried an A736V substitution in the Cdr transcription factor Tac1p. Tac1p A736V has been confirmed to confer azole resistance not only by regulating the expression of Cdr pumps but also by blocking morphogenesis in response to Hsp90 inhibitors (31). The upregulation of the *CDR2* gene was also found in two Clade 1-R-β, one Clade 1-R, and two Clade 1 isolates that were azole-NS/NWT. All of these isolates carried Tac1p substitutions, including H263Y, E461K, and R693K, which have previously been described (31, 32), as well as one novel substitution, L704F, which was detected here for the first time. No distinct change in the expression level of the *CDR1* or *CDR2* genes was observed in the azole-S/WT isolates that were examined. We also analyzed the connection between azole-resistance and unique LOH events that were found in the Clade 1-R and Clade 1-R-α isolates, including the LOH event that occurred on the chromosome R of all of the Clade 1-R-α isolates, but no positive correlation was found. Li et al. previously reported that the trisomy of chromosome R conferred azole resistance in C. albicans, but they also found that reduced susceptibility did not result from the overexpression of any known resistance-related genes in this region, and as such, researchers are still attempting to illustrate the underlying mechanisms (33).

Resistance to other antifungal agents, as per current criteria, was uncommon. One of the only two isolates that was nonsusceptible to echinocandins was also NS/NWT to azoles. As only limited classes of antifungal agents are available in clinical practice to date (3, 8), the emergence of multidrug-resistant C. albicans isolates should also be noted. With C. albicans, the predominant human-pathogenic fungal species, as an example, our study suggested that drug resistance in fungi could also be associated with phylogenetic lineages. It was revealed that over 66% of the azole-NS/NWT C. albicans strains belonged to the world’s largest population, namely, Clade 1. Furthermore, two nested subclades in Clade 1, namely, Clade 1-R and Clade 1-R-α, exhibited even higher proportions of isolates with azole-resistance. As azoles remain one of the most common antifungals used for the treatment of deadly fungal infections, concerns were raised. The further monitoring of the spread of these phylogenetic groups is warranted. In principle, the widespread use of azoles in clinical practice is a theoretical driver for resistance. However, we do not have data to indicate that this is so. More information is required to accurately evaluate this.

## MATERIALS AND METHODS

### Ethics statement.

This study was approved by the ethics committee of the National Institute for Communicable Disease Control and Prevention (ICDC), and it adhered to the guidelines of the Helsinki Declaration.

### Isolates involved in the genomic population study.

A total of 551 C. albicans genomes were used in the genomic population analysis. First, this set included 181 global C. albicans genomes that were from five continents and were previously published and reported by Ropars et al. (16). Of these isolates, 87.8% (159/181) were derived from humans, 10.5% (19/181) from the environment, and 1.1% (2/181) from animal sources.

In addition, there were a total of 370 C. albicans isolates, each from a unique (single) patient, that were collected from 33 cities (3 to 31 strains from each city) across China during the period from 2010 to 2016 as part of the national surveillance program for invasive fungal infections (the CHIF-NET study) (Fig. 3; Table S1; Table S11). The CHIF-NET study inclusion criteria have been described previously (34). These strains were mainly isolated from blood (80.8%, 299/370), ascitic fluid (8.1%, 30/370), pus (3.8%, 14/370), and bile (2.9%, 11/370).

The isolates were stored at −80°C at Peking Union Medical College Hospital, Beijing, China (PUMCH), until use. Before testing, the isolates were inoculated on CHROMagar *Candida* medium (Difco Laboratories, Detroit, MI, USA) and incubated at 28°C for 24 h. Species identification was confirmed via matrix-assisted laser desorption/ionization-time of flight mass spectrometry (MALDI-TOF MS, Vitek MS, bioMérieux, Marcyl’E’toile, France) and a sequence analysis of the rDNA internal transcribed spacer (ITS) regions of the strains (35, 36).

### Antifungal susceptibility of the involved strains.

The *in vitro* susceptibilities to nine antifungals (fluconazole, voriconazole, itraconazole, posaconazole, caspofungin, micafungin, anidulafungin, amphotericin B, and 5-flucytosine) were determined for the 370 isolates that were collected in China, using the Clinical and Laboratory Standards Institute (CLSI) broth microdilution method. The MIC results for fluconazole, voriconazole, caspofungin, micafungin, and anidulafungin were interpreted as susceptible (S), intermediate (I), susceptible-dose dependent (SDD), or resistant (R), using the clinical breakpoints (CBPs) in the CLSI guidelines, and the MICs for itraconazole, posaconazole, amphotericin B, and 5-flucytosine were interpreted as wild-type (WT) or non-wild-type (NWT), using epidemiological cutoff values (ECVs) (Table S10) (37–39). The quality control strains that were used were C. albicans SC5314, Candida krusei ATCC 6258, and Candida parapsilosis ATCC 22019. To reduce ambiguity, the resistant, intermediate, and SDD strains were described as nonsusceptible in this study.

The susceptibility data for the nine antifungal agents against the 370 isolates collected in China are detailed in Tables S1 and S2 as well as in Fig. S1. To more effectively explore whether the reduced antifungal susceptibility phenotype(s) were related to the development and expansion of certain phylogenetic clusters, azole-resistant isolates were selected preferentially in our study.

### Genome resequencing and quality filtering.

All of the fungal isolates were grown on potato dextrose agar at 28°C for 48 h. The DNA extraction was performed using a QIAamp DNA Mini Kit (Qiagen, Hilden, Germany). A paired-end library with an average insert size of 300 bp was prepared using a Nextera XT DNA Library Preparation Kit (Illumina, CA, USA) and was sequenced using an Illumina HiSeq X 10 platform with the PE150 (paired-end sequencing, 150 bp reads) sequencing mode. Raw FASTQ files were processed through a standard pipeline that consisted of low-quality read filtering through Trimmomatic (version 0.36) (40). The reads have been deposited at the NCBI Sequence Read Archive under BioProject ID PRJNA689765.

### Read mapping, variant identification, and filtering.

For each isolate, the reads were mapped to the C. albicans SC5314 reference genome (GenBank assembly accession: GCA_000182965.3) using the Burrows-Wheeler Aligner (BWA version 0.7.7) with the BWA-MEM algorithm (41). SAMtools version 1.6 (42) and Picard tools version 1.94 (http://broadinstitute.github.io/picard) were then used to filter, sort, and convert SAM files. Indel and variant calling were performed using the Genome Analysis Toolkit (GATK) (v.4.3.0.0), according to the GATK Best Practices (43). For each sample, variants were first called with the GATK HaplotypeCaller to create single-sample gVCFs with the option -emitRefConfidence GVCF, using the GVCF extension for the output file, and using Combine GVCFs to aggregate the GVCF files. The gVCFs were then jointly genotyped with the Genotype GVCFs function of GATK to produce a single multisample SNP file that contained data on every strain. Then, the SNPs and indels were filtered using the following parameters: VariantFiltration, QD < 2.0, ReadPosRankSum < −8.0, FS > 60.0, MQRankSum < −12.5, MQ < 40.0, and HaplotypeScore > 13.0. The sequencing depth was calculated using SAMtools version 1.6. The parameter -aa was added to count the sequencing depth at the reference, and then the average of all of the site depths in each sample was calculated.

### Loss of heterozygosity (LOH) analysis.

The joint calling method of GATK creates a complete genotyping matrix (.vcf format). This matrix of SNPs after filtering (see “Read mapping, variant identification, and filtering” above) included 741,793 sites across 551 isolates. To analyze the LOH patterns, the genetic state of each locus in each sample was coded to distinguish the loci that were homozygous for the haploid reference (−1), heterozygous SNPs (0), and homozygous SNPs for the nonreference state (1). We divided the genome into consecutive 10 kb windows and calculated the number of heterozygous SNPs in each window (with allele frequencies between 0.15 and 0.85). Then, we created a table that encompassed all 551 isolates from the VCF files, using custom scripts. The plots were generated using the R package ggplot2.

### Phylogenetic and population structure analysis.

IQ-TREE (v 1.6.12) was used to infer a maximum likelihood (ML) tree of the 551 isolates, using the data set of 741,793 confident SNPs and 1,000 bootstrap replicates (44). This study followed the midpoint rooting method that was used in a previous study (16), in which the root was set at the midpoint between the two most divergent isolates. The program GCTA (v 1.94.1) was used to conduct a principal components analysis that was based on the binary format files generated by PLINK (v1.9) (45). ADMIXTURE v1.3 was used to detect and quantify the number of populations and the degree of admixture in the 551 genomes (46). ADMIXTURE was run for K = 1 to K = 30, with 10 iterations per K. A cross-validation procedure was applied to infer the best number of ancestral populations (K). The K value with the lowest standard error of cross-validation was assumed to be the best indicator of the admixture pattern among populations. The strains assigned to Clade 1 and Cluster E in the previous analysis were submitted for a phylogeny and population structure analysis by IQ-TREE (v 1.6.12) and ADMIXTURE v1.3 to investigate the internal details of Clade 1 more clearly. The strain F0306, which had been assigned to group 6, which was adjacent to Clade 1 and Cluster E, was used as an outgroup in the ML tree of Clade 1 and Cluster E. The PCA of Clade 1 and Cluster E was also conducted via GCTA (v 1.94.1).

### Mutation analysis of genes associated with reduced antifungal susceptibility.

We reviewed published studies and summarized the C. albicans genes that may contribute to antifungal resistance (detail in Tables S1 and S5) (9–12). The mutations in these resistance-related genes were identified by comparing the studied isolates to the C. albicans SC5314 reference genome. A custom script was used to find the mutation site of each gene from the VCT file, according to the gene location information.

### Mutagenesis of the C. albicans *ERG11* gene and functional verification in the S. cerevisiae model.

A literature review was carried out to identify the previously verified *ERG11* gene mutations that caused an increase in the azole MICs (summarized in Table S5). However, previously characterized key substitutions, such as Y132H, could also be found among the azole-susceptible clinical isolates in this study. In addition, a novel Erg11p substitution, namely, L301S, was observed in an azole-NS/NWT isolate. Therefore, we repeated the functional verification of the Erg11p substitutions found in this study in an S. cerevisiae model.

A complete *ERG11* open reading frame adjacent to the upstream and downstream regions was amplified from C. albicans SC5314 and ligated to the pBM16A vector, using a pBM16A Toposmart Cloning Kit (Biomed, Beijing, China), according to the manufacturer’s instructions. The recombinant plasmid obtained, named pBM16A-*ERG11*^WT^, was enriched in Escherichia coli DH-5α (Transgene, China) and sequenced from both directions using an ABI3730 sequencer (ABI, Foster City, USA) to confirm the presence of *ERG11* wild-type sequences (100% identical to GenBank accession GCA_000182965.3). Site-directed mutagenesis was then performed on pBM16A-*ERG11*^WT^, using a Q5 Site-directed Mutagenesis Kit (NEB, USA), according to the manufacturer’s instructions. The amplification primers were designed at 428 bp upstream and 212 bp downstream of the open reading frame of the *ERG11* gene. The primer sequences are shown in Table S3. The primers used for the mutagenesis of each target SNP were designed by the NEBaseChanger online software (http://nebasechanger.neb.com), and they are shown in Table S3. A total of nine conjugated pBM16A plasmids with single nucleotide mutations, namely, pBM16A-*ERG11*^A114S^, pBM16A-*ERG11*^T123I^, pBM16A-*ERG11*^Y132H^, pBM16A-*ERG11*^K143R^, pBM16A-*ERG11*^F145L^, pBM16A-*ERG11*^Y257H^, pBM16A-*ERG11*^L301S^, pBM16A-*ERG11*^S405F^, and pBM16A-*ERG11*^G448E^, were obtained. Furthermore, *ERG11* gene sequences with double mutations, which could be observed from clinical strains, were induced in a second round of mutagenesis, using the pBM16A-*ERG* plasmid with single mutations as the template. Four pBM16A plasmids, namely, pBM16A-*ERG11*^A114S, Y257H^, pBM16A-*ERG11*^T123I, Y132H^, pBM16A-*ERG11*^Y132H, S405F^, and pBM16A-*ERG11*^Y132H, G448E^, were successfully acquired.

The next steps proceeded according to a protocol published by Fan et al. (19), with some modifications. Briefly, the wild-type *ERG11* gene sequence and its single- or double-SNP mutants were amplified from the corresponding pBM16A-*ERG11* plasmids, using an In-Fusion Cloning primer set (Table S3). They were then cloned into the KpnI site of the shuttle vector pYES2/CT (Biofeng Biotechnology Co., Ltd., Shanghai, China), using an In-Fusion Cloning Kit (TaKaRa Bio Inc., USA). The pYES2/CT plasmid contained the inducible promoter *GAL1*, and galactose was able to induce stable gene expression. The pYES2/CT plasmid contained both the ampicillin resistance gene and the *URA3* gene as selective markers. It also contained the inducible promoter *GAL1*, where galactose induces stable target gene expression, while glucose blocks expression. The ligated plasmids were enriched again in E. coli DH5α and were transformed into S. cerevisiae W303-1a using a Frozen-EZ Yeast Transformation II Kit (Zymo Research, Irvine, CA, USA). They were then selected on synthetic dropout-uracil agar plates that contained glucose. Dropout-uracil liquid medium containing 2% galactose and 2% raffinose was used to induce heterologous expression of the C. albicans *ERG11* gene in S. cerevisiae, and these strains were tested for antifungal susceptibility to fluconazole via the Etest (Autobio, Zhengzhou, China) methodology. Each MIC was read after incubation at 30°C for 48 h and was determined at the concentration at which the colony size was apparently reduced (80% growth inhibition), following the manufacturer's instructions.

### Exploring gene expression changes associated with azole susceptibility in Clade 1/Cluster E isolates.

The gene expression levels of the drug target *ERG11* gene, drug efflux pump-encoding genes *CDR1*, *CDR2*, *MDR1*, and *FLU1*, and mitochondrial *CYTb* gene regulating respiratory status were tested among 33 selected Clade 1/Cluster E isolates in this study. These 33 isolates involved all 9 Clade 1-R-α isolates and 9 Clade 1-R-β isolates, as well as 6 azole-S/WT and 6 azole-NS/NWT isolates that were assigned to Clade 1/Clade 1-R (but not Clade 1-R-α/β), as well as 3 azole-S/WT isolates in Cluster E. Briefly, all of the selected fungal isolates were grown in yeast peptone dextrose medium at 28°C for 48 h. RNA extraction was performed using an RNeasy Mini Kit (Qiagen, Hilden, Germany). Real-time quantitative PCR was used to measure the expression levels of the *ERG11*, *CDR1*, *CDR2*, *MDR1*, *FLU1*, and *CYTb* genes, with the *ACT1* gene being used as an internal control, using previously published primers and amplification conditions (detailed in Table S3) (30, 47, 48). SC5314 was used as the reference strain. The gene expression levels were evaluated using the comparative CT method (49).

### Data availability.

The data generated and analyzed during this study are included in this published article and in its additional information files. Further data sets that were used and analyzed during the current study are available from the corresponding authors upon reasonable request.
