# Supplementary material for: Emergence of Antifungal Resistant Subclades in the Global Predominant Phylogenetic Population of Candida albicans
Source: Microbiol Spectr. 2023 Jan 26;11(1):e03807-22. doi: 10.1128/spectrum.03807-22 (PMC9927326; doi:10.1128/spectrum.03807-22)
Supplement: Supplemental file 1 — Supplemental material. Download spectrum.03807-22-s0001.pdf, PDF file, 7.2 MB [file spectrum.03807-22-s0001.pdf]

Supplemental materials

Supplemental materials include 10 figures and 11 tables of data.

Fig. S01 *In vitro* susceptibility of 370 *C. albicans* clinical strains to nine antifungal agents. Abbreviations: MIC, minimum inhibitory concentration; S, susceptible; SDD, susceptible-dose dependent; I, intermediate; R, resistant; WT, wild-type; NWT, non-wild-type.

Fig. S02 Mean genome sequencing depth of 370 *C. albicans* clinical strains involved in this study and 181 global *C. albicans* genomes.

Fig. S03 Phylogenetic relationship and population structure of 370 *C. albicans* clinical isolates in China together with 181 global *C. albicans* genomes. The left half showed maximum likelihood tree generated based on whole-genome SNPs, and the right half showed population structures of *C. albicans* (the number of populations [K] from 2 to 25 was exhibited).

Fig. S04 Loss-of-heterozygosity (LOH) analysis for all clinical isolates from China except for those belonged to Clade 1 (which were shown in Fig. 3). Density of heterozygous SNPs was calculated using *C. albicans* SC5314 as reference and shown in 10 kb windows. Each row represented a *C. albicans* strain.

Fig. S05 Principal components analysis (PCA) of Clade 1 and Cluster E isolates.

Fig. S06 Minimum inhibitory concentration (MIC) distribution of fluconazole-susceptible strains within Clade 1-R-β versus fluconazole-susceptible non-Clade 1-R-β isolates.

Fig. S07 Minimum inhibitory concentration (MIC) distribution of strains carrying the *FUR1* gene mutation causing the R101C substitution versus those not carrying the mutation.

Fig. S08 Cross-Validation error as a function of K for admixture analysis. (A) Cross-Validation error of all 551 isolates. (B) Cross-Validation error of 106 isolates assigned to Clade 1 and adjacent Cluster E

Fig. S09 Fluconazole MIC fold-Change in the heterologous expression model of *S. cerevisiae* with important Ergl1p substitution(s).

Fig. S10 Heatmap of pairwise SNP distances based on the absolute distance of SNPs.

Fig. S11 Phylogenetic relationship and population structure of Clade 1 and adjacent Cluster E. The left half showed maximum likelihood tree generated based on whole-genome SNPs, and the right half showed population structures of *C. albicans* (the number of populations [K] from 2 to 9 was exhibited).

Table S01. A comprehensive summary for characteristics of 370 *C. albicans* isolates collected in this study and 181 global genomes involved as comparators, including each isolate's genomic data source, phylogenetic characteristics, antifungal susceptibility phenotypes, geographic and clinical origins, and mutations observed in genes associated with antifungal resistance.

Table S02. Nucleotide diversity and intermediate (I)/susceptible dose-dependent (SDD)/resistant (R)/non-wild-type (NWT) rate of *C. albicans* isolates within different clonal populations.

Table S03. List of primers used in this study.

Table S04. List of predicted genes on chromosome regions where unique loss-of-heterozygosity (LOH) events were observed within genetic populations Clade 1-R and Clade 1-R-α.

Table S05. Association between Erg11p substitutions detected in *C. albicans* and azole susceptibilities, using *S. cerevisiae* and/or *C. albicans* models, from literature review and in this study.

Table S06. Relative proportion of fluconazole-NS isolates in major phylogenetic clades detected from China in the study, versus that reported by Odds *et al.* from a global *C. albicans* collection.

Table S07. Changes in the expression levels of important azole-NS/NWT-related genes amongst 33 selected Clade 1/Cluster E strains, and their correlation with azole susceptibility phenotypes, *ERG11* genotypes and important substitutions identified in Tac1p.

Table S08. Review of previous literature reporting *C. albicans* isolates carrying Erg11p substitutions A114S and Y257H.

Table S09. Proportion of Clade 1-R and Clade 1-R-α isolates detected in different years.

Table S10. Clinical breakpoints (CBPs) and epidemiological cutoff values (ECVs) for interpreting antifungal susceptibility testing results of *C. albicans* isolates applied in this study.

Table S11. Summary statistics of 370 *C. albicans* isolates collected in this study.

Fig. S1

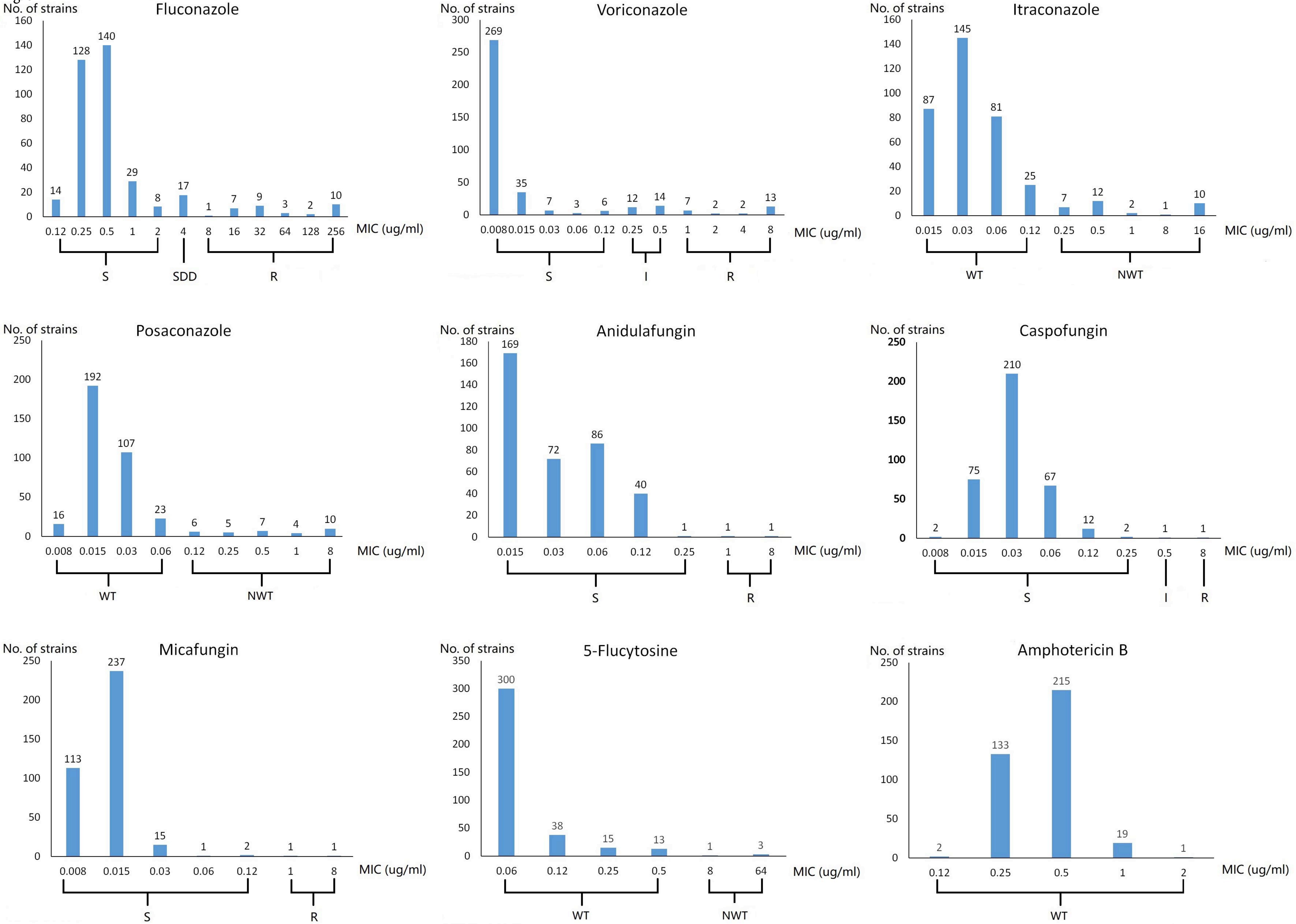

Fluconazole
No. of strains
0 20 40 60 80 100 120 140 160
14 128 140 29 8 17 1 7 9 3 2 10
0.12 0.25 0.5 1 2 4 8 16 32 64 128 256
MIC (ug/ml)
S
SDD
R
Voriconazole
No. of strains
0 50 100 150 200 250 300
269 35 7 3 6 12 14 7 2 2 13
0.008 0.015 0.03 0.06 0.12 0.25 0.5 1 2 4 8
MIC (ug/ml)
S
I
R
Itraconazole
No. of strains
0 20 40 60 80 100 120 140 160
87 145 81 25 7 12 2 1 10
0.015 0.03 0.06 0.12 0.25 0.5 1 8 16
MIC (ug/ml)
WT
NWT
Posaconazole
No. of strains
0 50 100 150 200 250
16 192 107 23 6 5 7 4 10
0.008 0.015 0.03 0.06 0.12 0.25 0.5 1 8
MIC (ug/ml)
WT
NWT
Anidulafungin
No. of strains
0 20 40 60 80 100 120 140 160 180
169 72 86 40 1 1 1
0.015 0.03 0.06 0.12 0.25 1 8
MIC (ug/ml)
S
R
Caspofungin
No. of strains
0 50 100 150 200 250
2 75 210 67 12 2 1 1
0.008 0.015 0.03 0.06 0.12 0.25 0.5 8
MIC (ug/ml)
S
I
R
Micafungin
No. of strains
0 50 100 150 200 250
113 237 15 1 2 1 1
0.008 0.015 0.03 0.06 0.12 1 8
MIC (ug/ml)
S
R
5-Flucytosine
No. of strains
0 50 100 150 200 250 300 350
300 38 15 13 1 3
0.06 0.12 0.25 0.5 8 64
MIC (ug/ml)
WT
NWT
Amphotericin B
No. of strains
0 50 100 150 200 250
2 133 215 19 1
0.12 0.25 0.5 1 2
MIC (ug/ml)
WT

Fig. S2

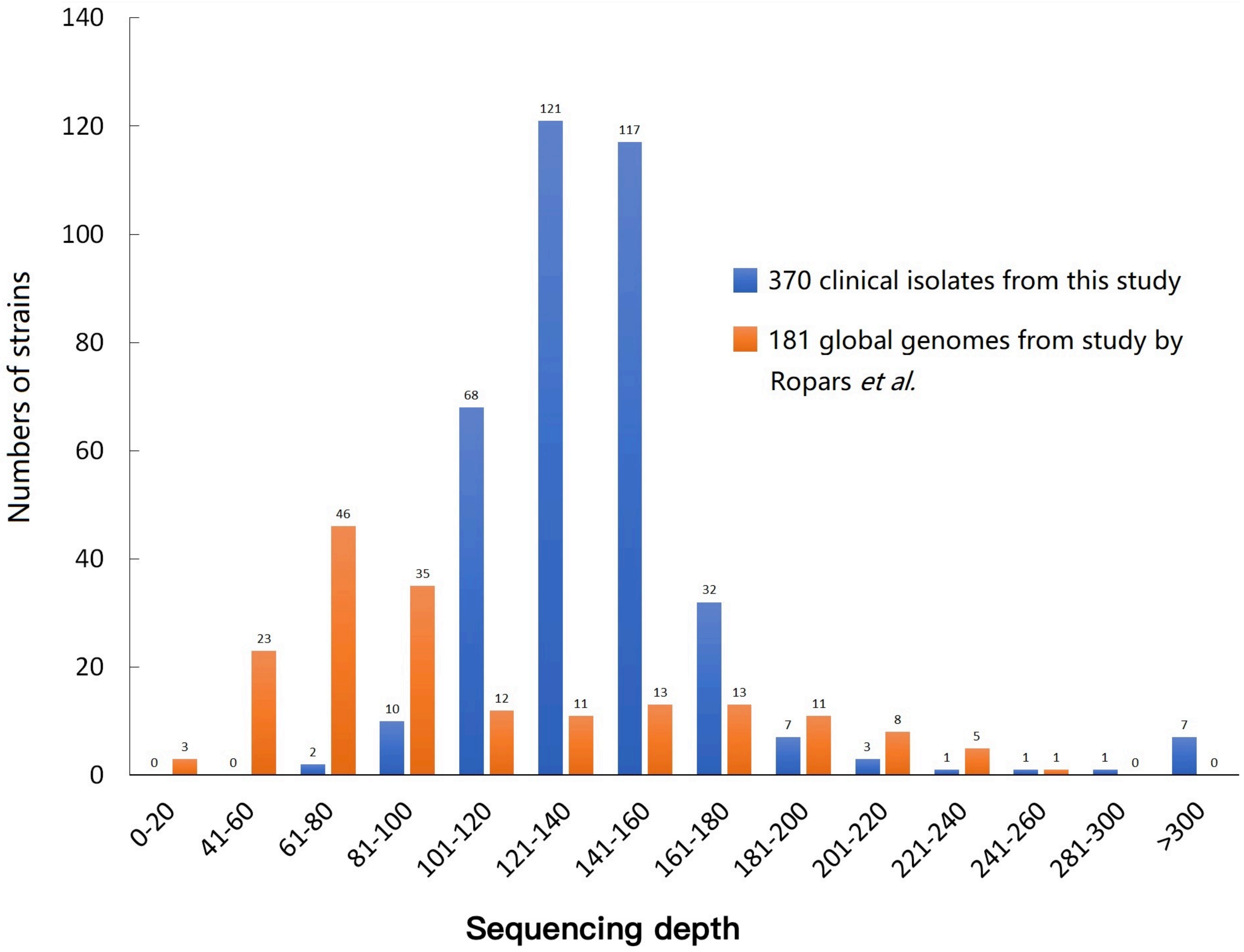

140
120
100
80
60
40
20
0
Numbers of strains
Sequencing depth
0-20
41-60
61-80
81-100
101-120
121-140
141-160
161-180
181-200
201-220
221-240
241-260
281-300
>300
0
3
0
23
2
46
10
35
68
12
121
11
117
13
32
13
7
11
3
8
1
5
1
1
1
0
7
0
370 clinical isolates from this study
181 global genomes from study by Ropars *et al.*

Fig. S3

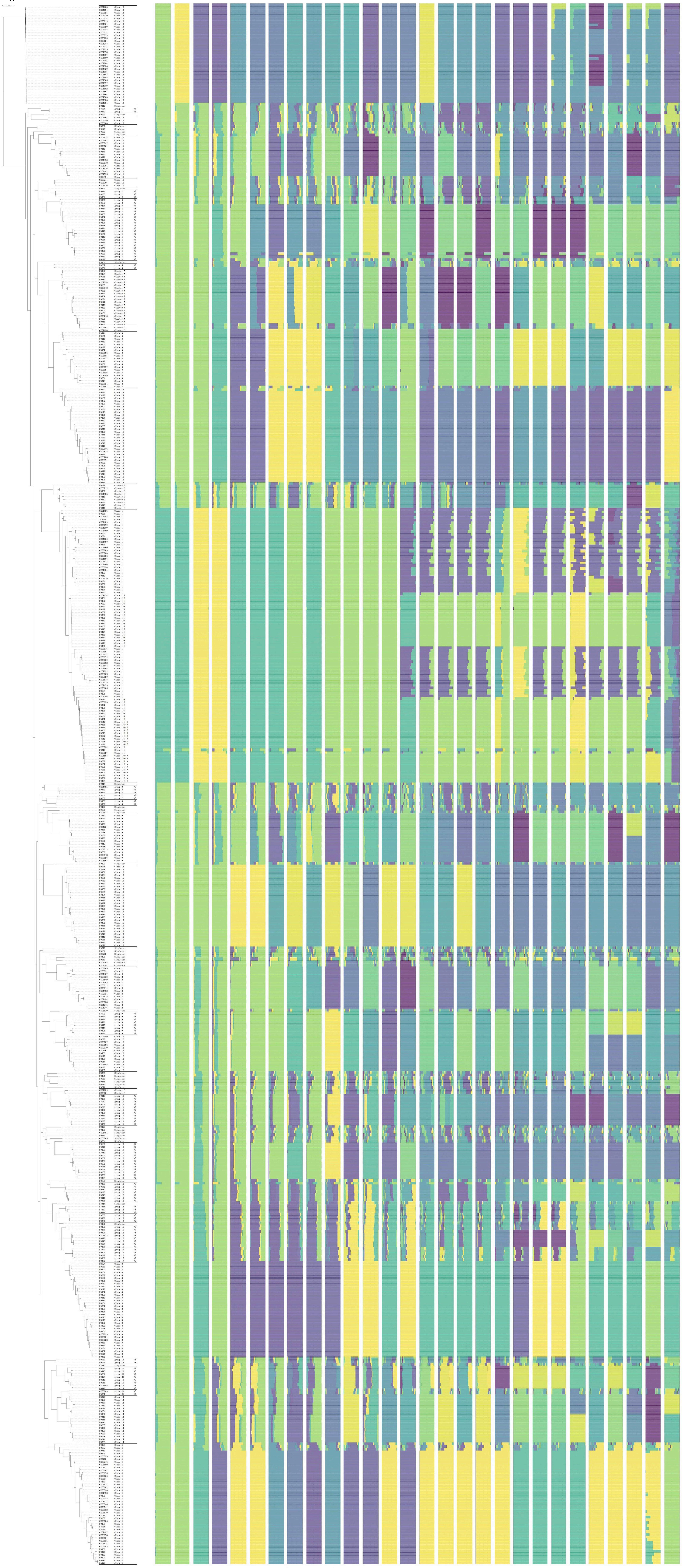

※, novel clonal populations identified in our study.

Population structure of 370 *C.albicans* clincal isolates from this study and 181 global *C.albicans* genomes.
The number of population (K) from 2 (left column) to 30 (right column) were shown.

Fig. S4

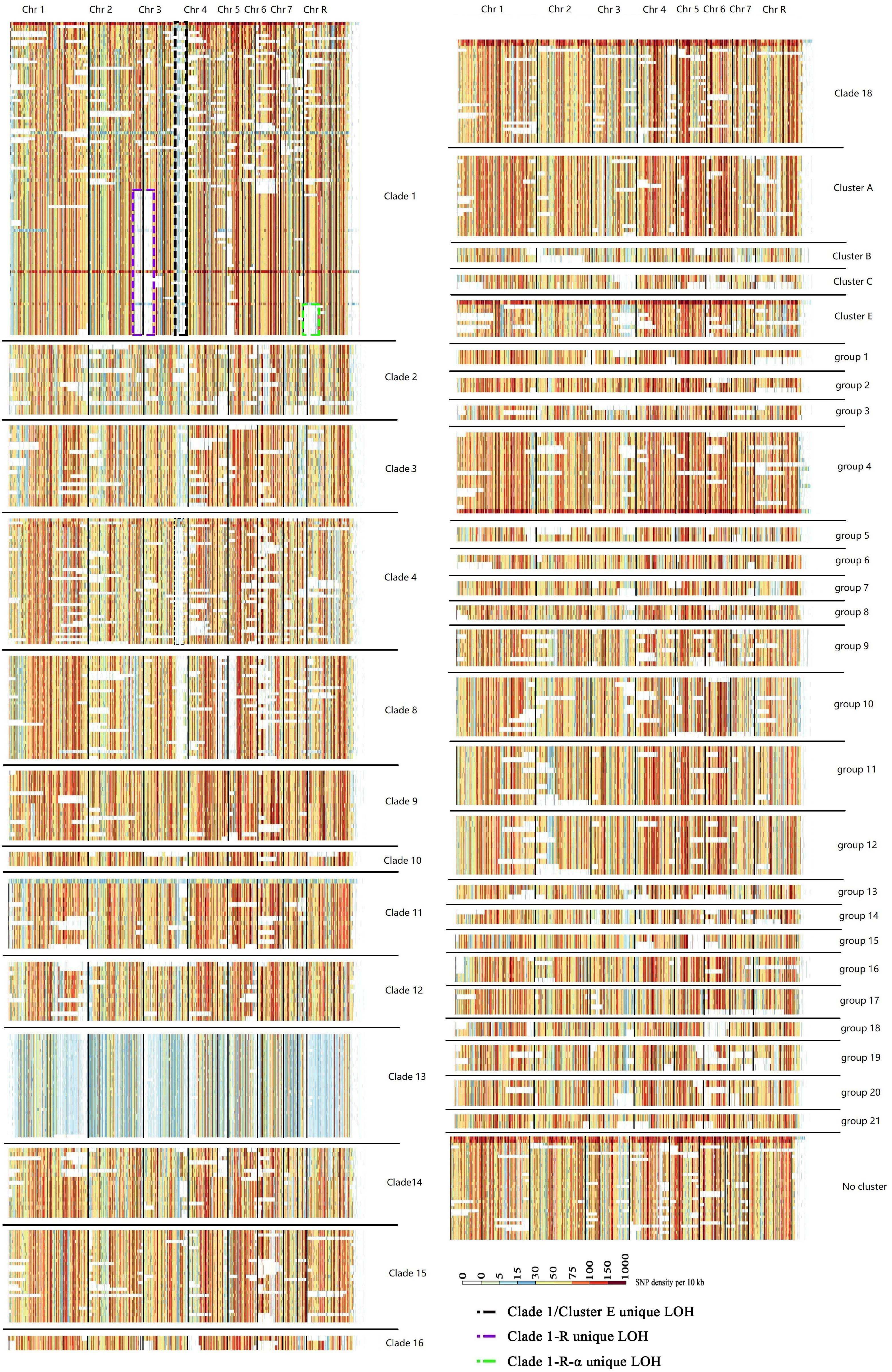
Chr 1
Chr 2
Chr 3
Chr 4
Chr 5
Chr 6
Chr 7
Chr R
Clade 1
Clade 2
Clade 3
Clade 4
Clade 8
Clade 9
Clade 10
Clade 11
Clade 12
Clade 13
Clade14
Clade 15
Clade 16
Clade 18
Cluster A
Cluster B
Cluster C
Cluster E
group 1
group 2
group 3
group 4
group 5
group 6
group 7
group 8
group 9
group 10
group 11
group 12
group 13
group 14
group 15
group 16
group 17
group 18
group 19
group 20
group 21
No cluster
0 0 5 15 30 50 75 100 150 1000
SNP density per 10 kb
Clade 1/Cluster E unique LOH
Clade 1-R unique LOH
Clade 1-R-α unique LOH

Fig. S5

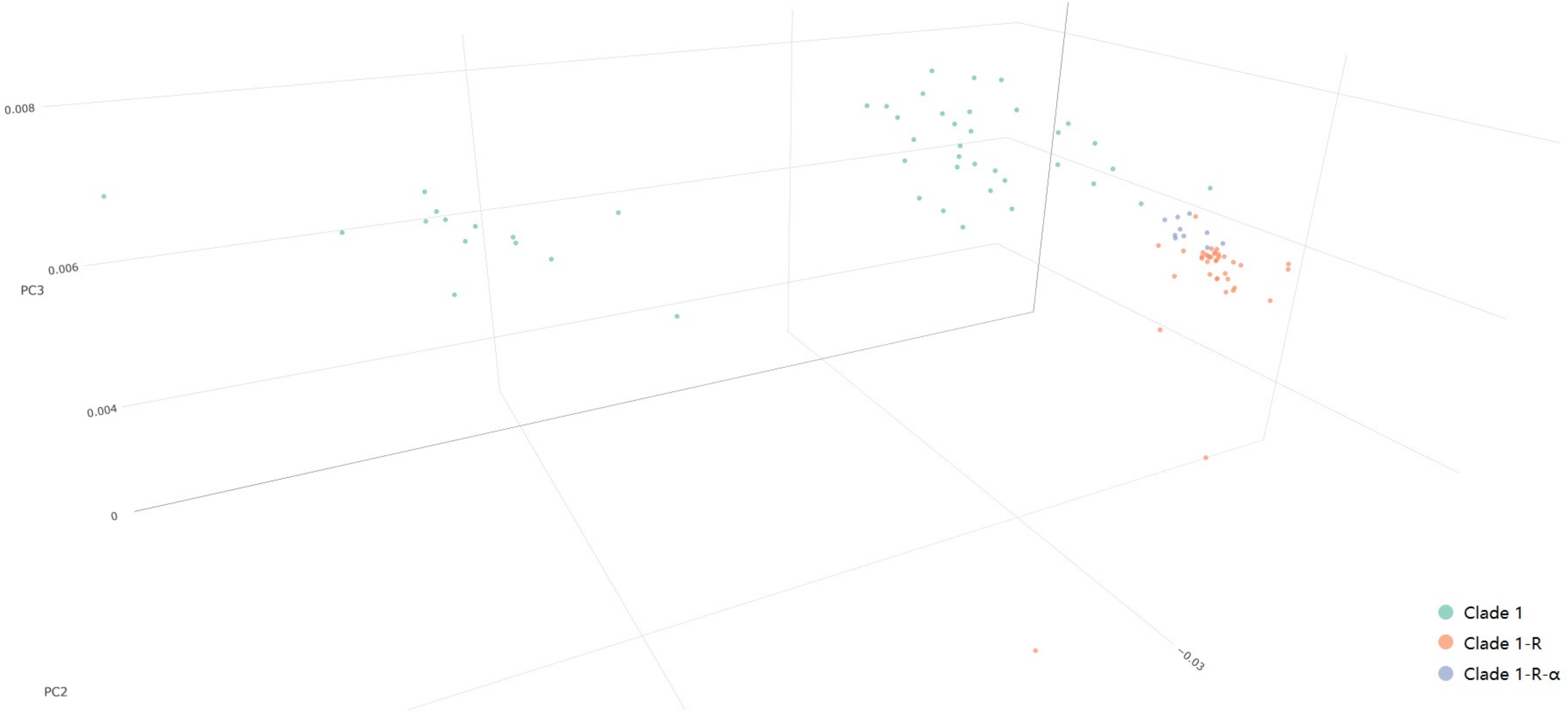

Fig. S6

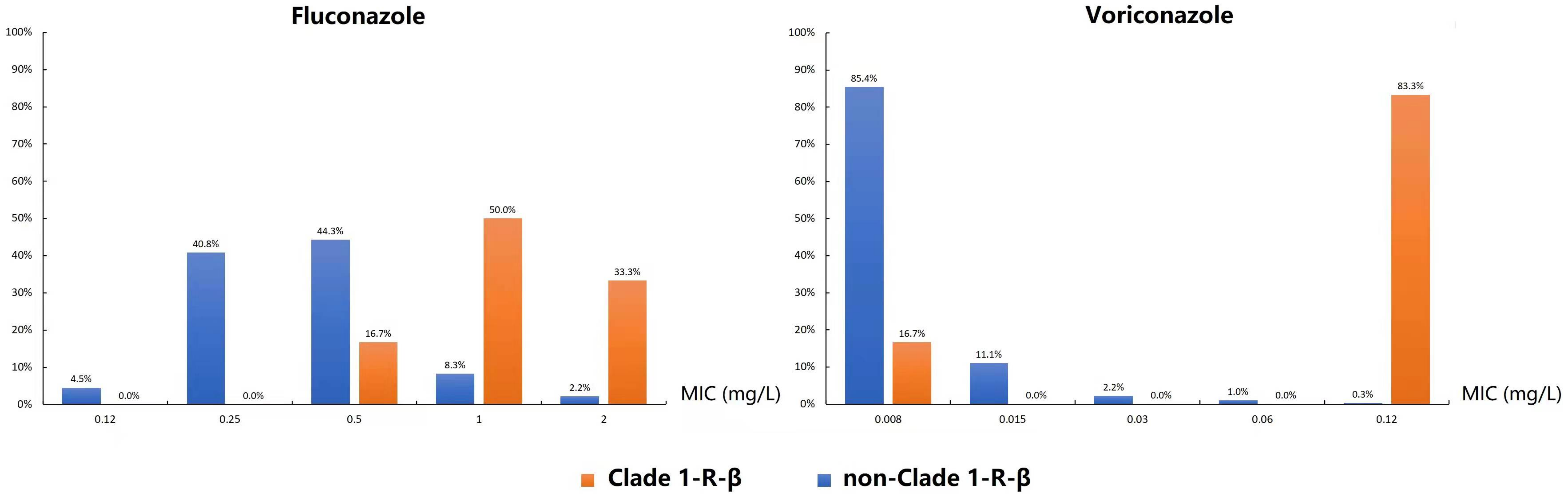

Fluconazole
100%
90%
80%
70%
60%
50%
40%
30%
20%
10%
0%
4.5%
0.0%
40.8%
0.0%
44.3%
16.7%
8.3%
50.0%
2.2%
33.3%
0.12
0.25
0.5
1
2
MIC (mg/L)
Voriconazole
100%
90%
80%
70%
60%
50%
40%
30%
20%
10%
0%
85.4%
16.7%
11.1%
0.0%
2.2%
0.0%
1.0%
0.0%
0.3%
83.3%
0.008
0.015
0.03
0.06
0.12
MIC (mg/L)
Clade 1-R-β
non-Clade 1-R-β

Fig. S7

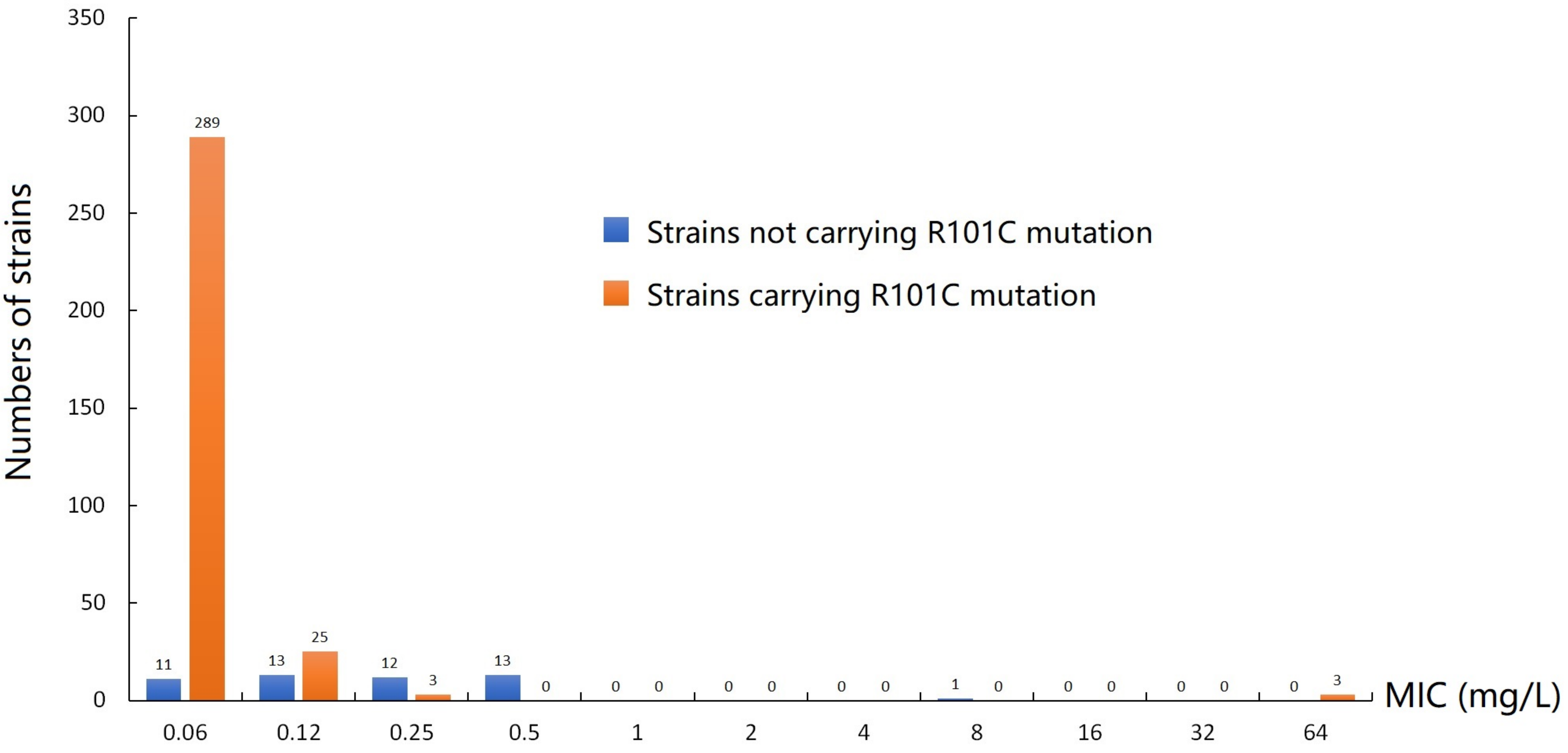

Numbers of strains
350
300
250
200
150
100
50
0
289
11
13
25
12
3
13
0
0
0
0
0
0
0
1
0
0
0
0
0
0
3
0.06
0.12
0.25
0.5
1
2
4
8
16
32
64
MIC (mg/L)
Strains not carrying R101C mutation
Strains carrying R101C mutation

Fig. S8

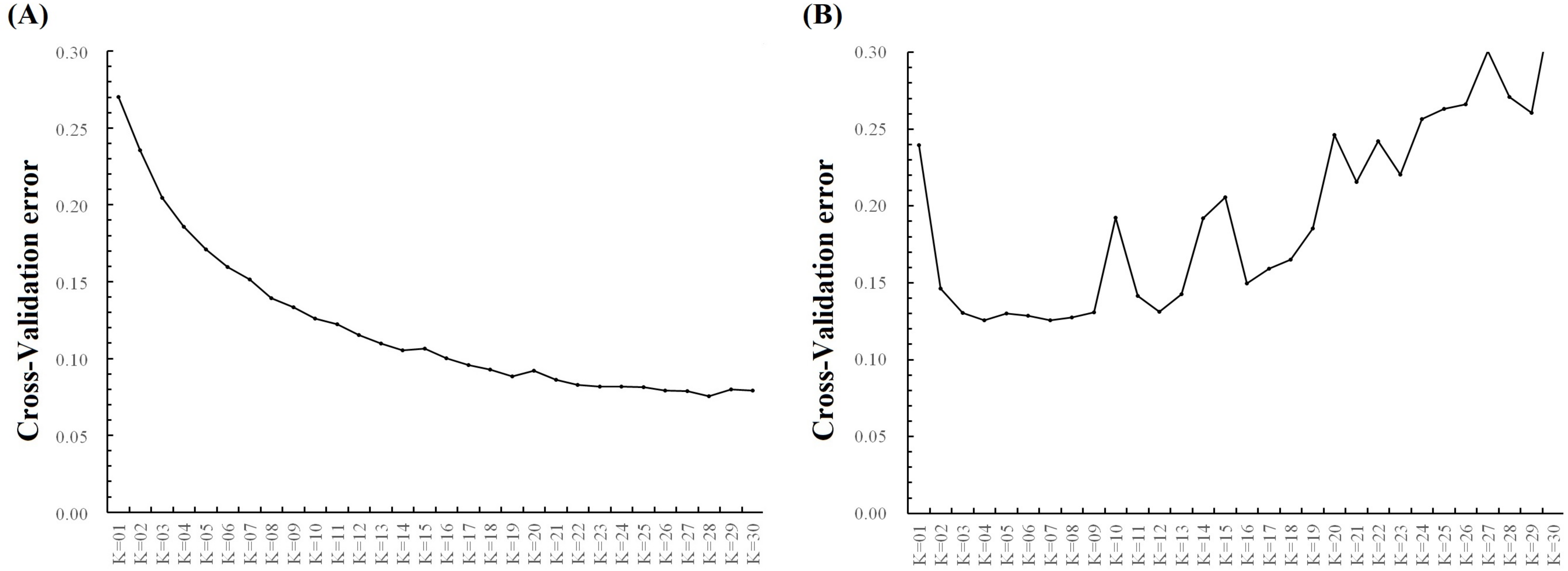
(A)
(B)
Cross-Validation error
0.30
0.25
0.20
0.15
0.10
0.05
0.00
K=01
K=02
K=03
K=04
K=05
K=06
K=07
K=08
K=09
K=10
K=11
K=12
K=13
K=14
K=15
K=16
K=17
K=18
K=19
K=20
K=21
K=22
K=23
K=24
K=25
K=26
K=27
K=28
K=29
K=30

Fig. S9

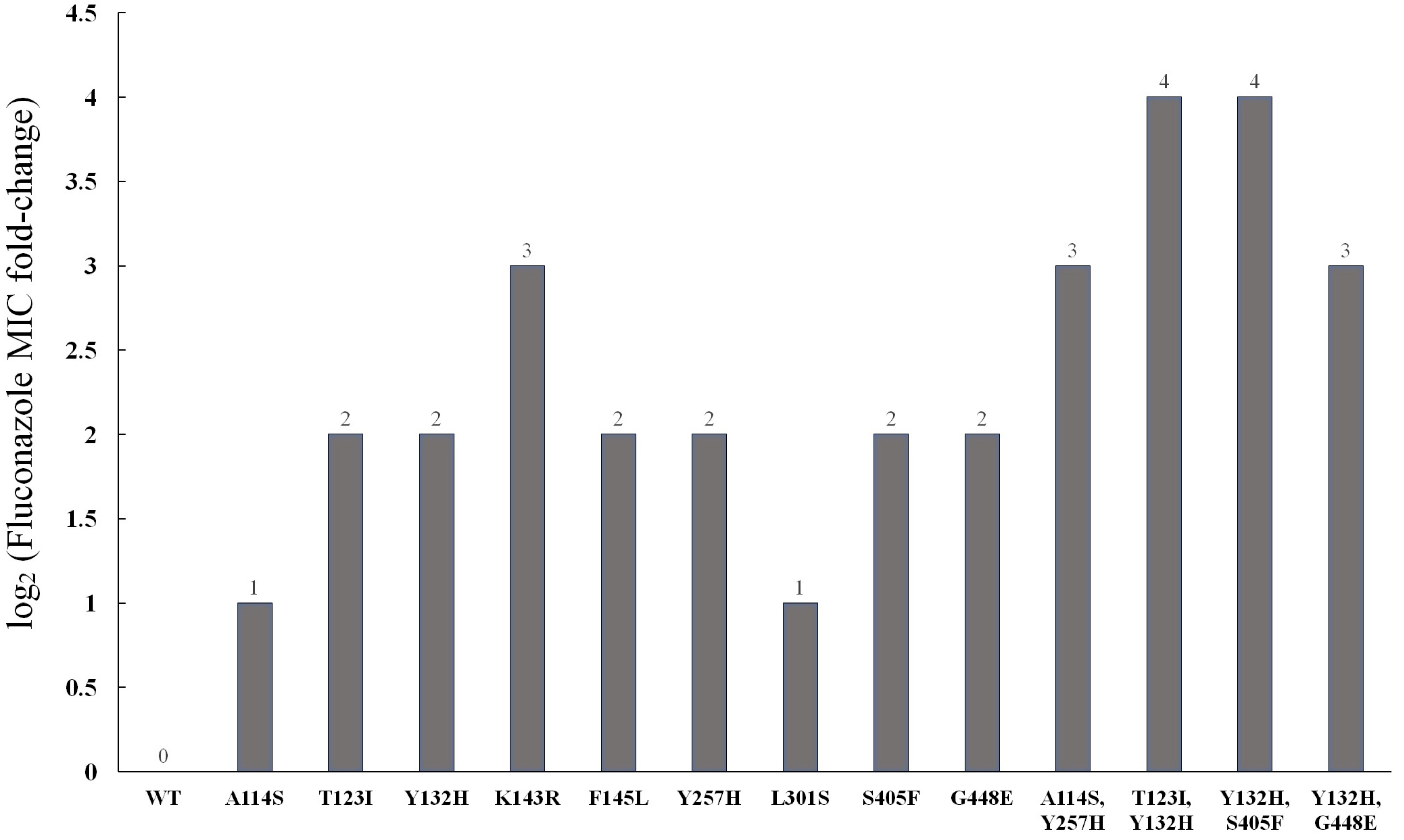

log2 (Fluconazole MIC fold-change)
0
0.5
1
1.5
2
2.5
3
3.5
4
4.5
0
1
2
2
3
2
2
1
2
2
3
4
4
3
WT
A114S
T123I
Y132H
K143R
F145L
Y257H
L301S
S405F
G448E
A114S, Y257H
T123I, Y132H
Y132H, S405F
Y132H, G448E

Fig. S10

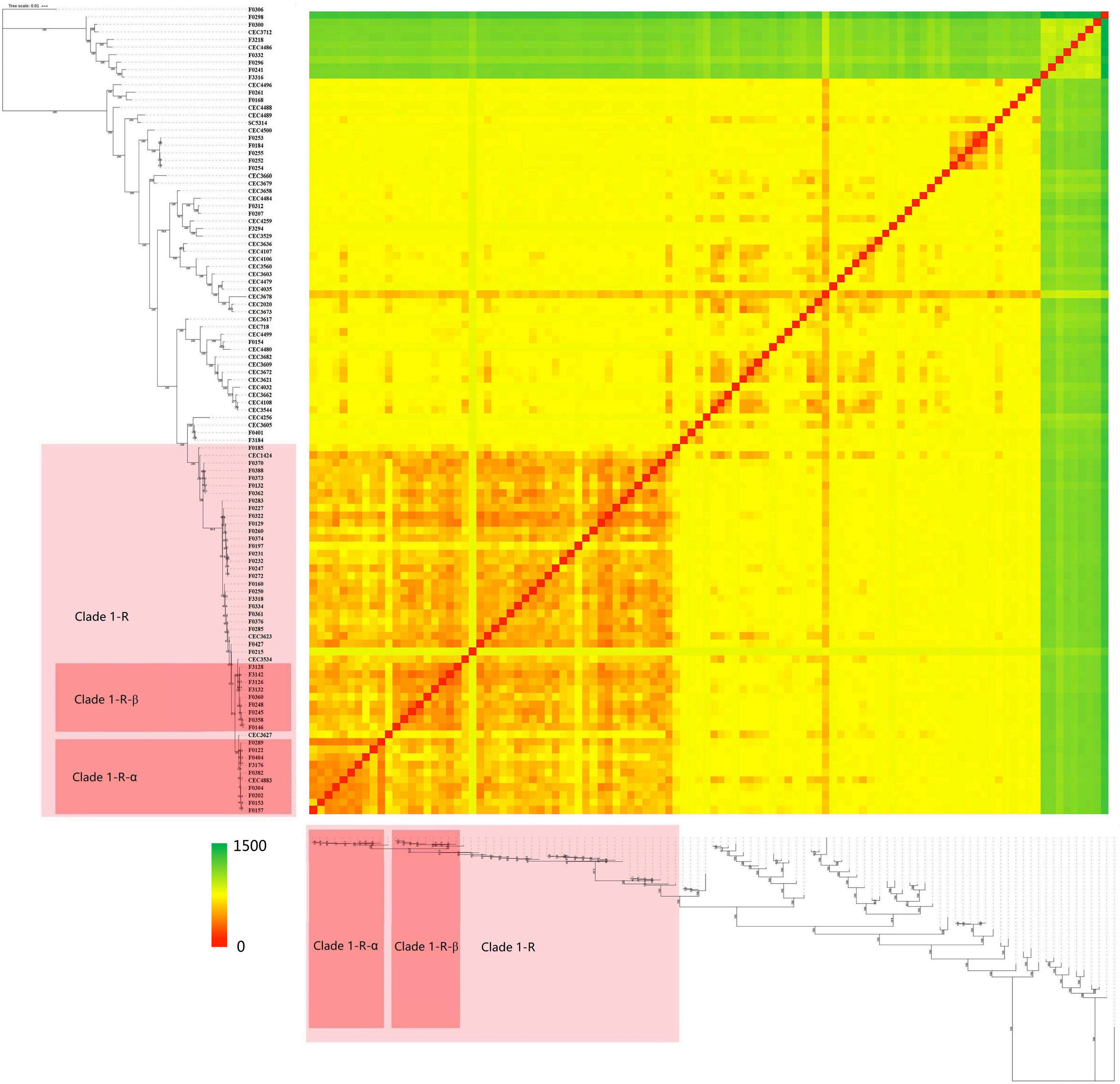

Tree scale: 0.01
F0306
F0298
F0300
CEC3712
F3218
CEC4486
F0332
F0296
F0241
F3316
CEC4496
F0261
F0168
CEC4488
CEC4489
SC5314
CEC4500
F0253
F0184
F0255
F0252
F0254
CEC3660
CEC3679
CEC3658
CEC4484
F0312
F0207
CEC4259
F3294
CEC3529
CEC3636
CEC4107
CEC4106
CEC3560
CEC3603
CEC4479
CEC4035
CEC3678
CEC2020
CEC3673
CEC3617
CEC718
CEC4499
F0154
CEC4480
CEC3682
CEC3609
CEC3672
CEC3621
CEC4032
CEC3662
CEC4108
CEC3544
CEC4256
CEC3605
F0401
F3184
F0185
CEC1424
F0370
F0388
F0373
F0132
F0362
F0283
F0227
F0322
F0129
F0260
F0374
F0197
F0231
F0232
F0247
F0272
F0160
F0250
F3318
F0334
F0361
F0376
F0285
CEC3623
F0427
F0215
CEC3534
F3128
F3142
F3126
F3132
F0360
F0248
F0245
F0358
F0146
CEC3627
F0289
F0122
F0404
F3176
F0382
CEC4883
F0304
F0202
F0153
F0157
Clade 1-R
Clade 1-R-β
Clade 1-R-α
1500
0
Clade 1-R-α
Clade 1-R-β
Clade 1-R

Fig. S11

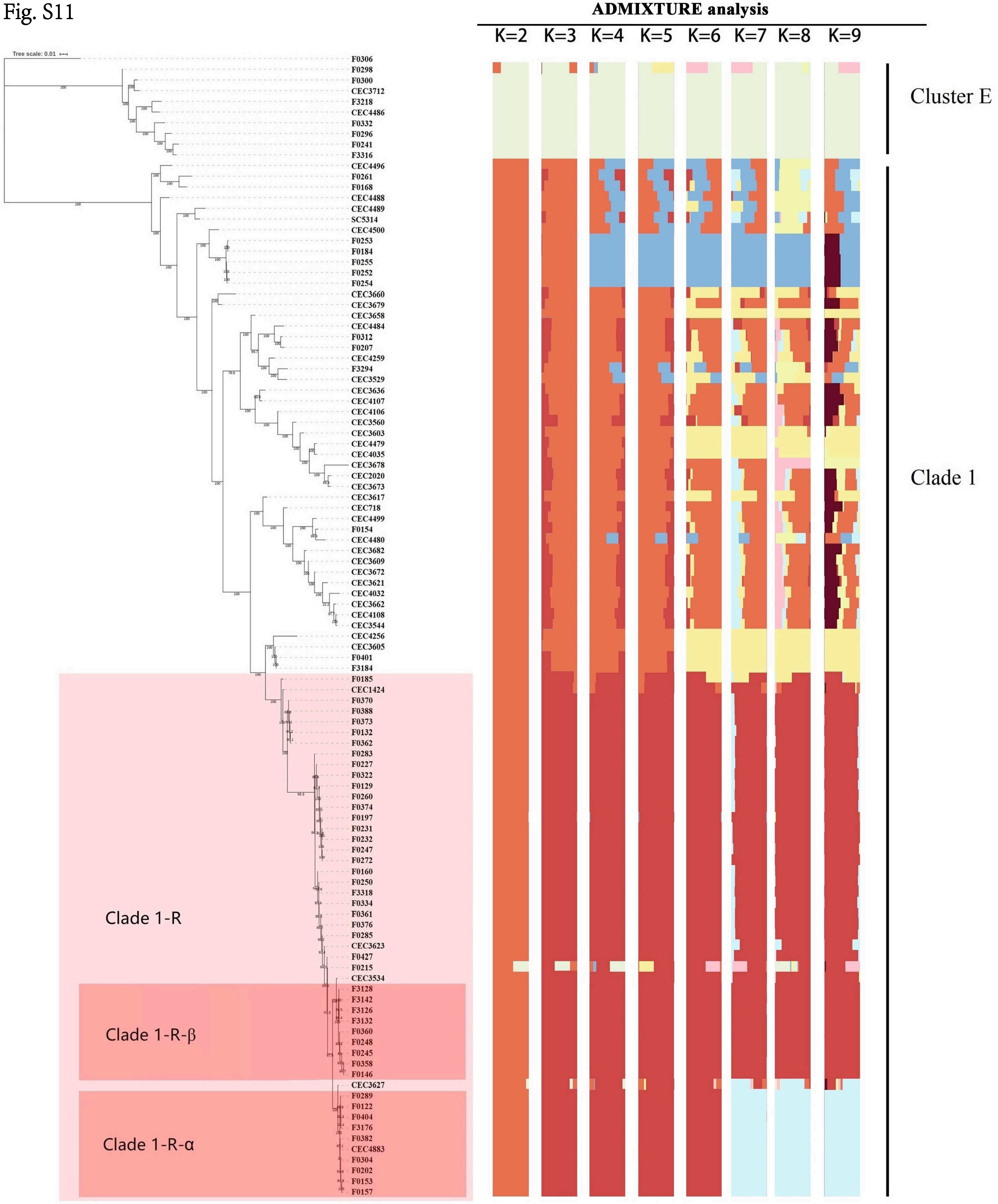
ADMIXTURE analysis
K=2
K=3
K=4
K=5
K=6
K=7
K=8
K=9
Tree scale: 0.01
Cluster E
Clade 1
Clade 1-R
Clade 1-R-β
Clade 1-R-α
F0306
F0298
F0300
CEC3712
F3218
CEC4486
F0332
F0296
F0241
F3316
CEC4496
F0261
F0168
CEC4488
CEC4489
SC5314
CEC4500
F0253
F0184
F0255
F0252
F0254
CEC3660
CEC3679
CEC3658
CEC4484
F0312
F0207
CEC4259
F3294
CEC3529
CEC3636
CEC4107
CEC4106
CEC3560
CEC3603
CEC4479
CEC4035
CEC3678
CEC2020
CEC3673
CEC3617
CEC718
CEC4499
F0154
CEC4480
CEC3682
CEC3609
CEC3672
CEC3621
CEC4032
CEC3662
CEC4108
CEC3544
CEC4256
CEC3605
F0401
F3184
F0185
CEC1424
F0370
F0388
F0373
F0132
F0362
F0283
F0227
F0322
F0129
F0260
F0374
F0197
F0231
F0232
F0247
F0272
F0160
F0250
F3318
F0334
F0361
F0376
F0285
CEC3623
F0427
F0215
CEC3534
F3128
F3142
F3126
F3132
F0360
F0248
F0245
F0358
F0146
CEC3627
F0289
F0122
F0404
F3176
F0382
CEC4883
F0304
F0202
F0153
F0157
